# Analysing volatile organic compounds (VOCs) with SERS by bubbling into colloidal nanoparticles

**DOI:** 10.1039/d6an00673f

**Published:** 2026-08-17

**Authors:** Amy Colleran, Nigel Gotts, Stephanie Murray, Royston Goodacre

**Affiliations:** a Centre for Metabolomics Research, Department of Biochemistry, Cell and Systems Biology, Institute of Systems, Molecular and Integrative Biology, University of Liverpool Crown St. Liverpool L69 7ZB UK roy.goodacre@liverpool.ac.uk; b Unilever Research and Development Port Sunlight Bebington CH63 3JW UK

## Abstract

Surface-enhanced Raman scattering (SERS) enables real-time analysis of volatile organic compounds (VOCs) and other gaseous molecules using portable Raman devices, offering faster measurements compared to GC-MS. To facilitate this, numerous solid SERS substrates have been developed and applied across multiple fields. However, they often require complex fabrication due to poor adsorption of gases onto solid surfaces leading to high production costs and inconsistencies in sensitivity and reproducibility. In contrast, colloidal nanoparticles are less expensive to produce and established facile synthesis methods help their broader implementation. In this study a simple approach has been conceptualised whereby gaseous analytes are bubbled directly into colloidal nanoparticles. This was achieved by pumping air into a flask containing the volatile analyte, which in turn was bubbled into a vial containing colloidal nanoparticles and measured with a portable Raman spectrometer. Initially, 3-mercapto-hexanol (3MH) was successfully measured using this method. 3MH was chosen due to the method originally being developed to measure thiols associated to underarm odour. Subsequently, 3MH was used to optimize the air flow rate by testing at eight different flow rates. The results demonstrated that flow rate influenced the timing of analyte spectral appearance as seen by the rate of change in peak height at 634 cm^−1^ for 3MH, with the most reproducible results observed at 50 mL min^−1^. The technique was further evaluated with two other analytes: 2-methyl-3-mercapto-pentanol (2M3MP) and indole. The latter needed the addition of an aggregating agent (NaCl) to the nanoparticles. These findings indicate that this approach offers a promising alternative for detecting gaseous analytes using colloidal nanoparticles.

## Abstract 2

A simple and novel surface-enhanced Raman scattering (SERS) technique has been developed for the detection of volatile organic compounds (VOCs) by bubbling VOCs directly into colloidal nanoparticles and measuring with a portable Raman spectrometer.

## Graphical abstract

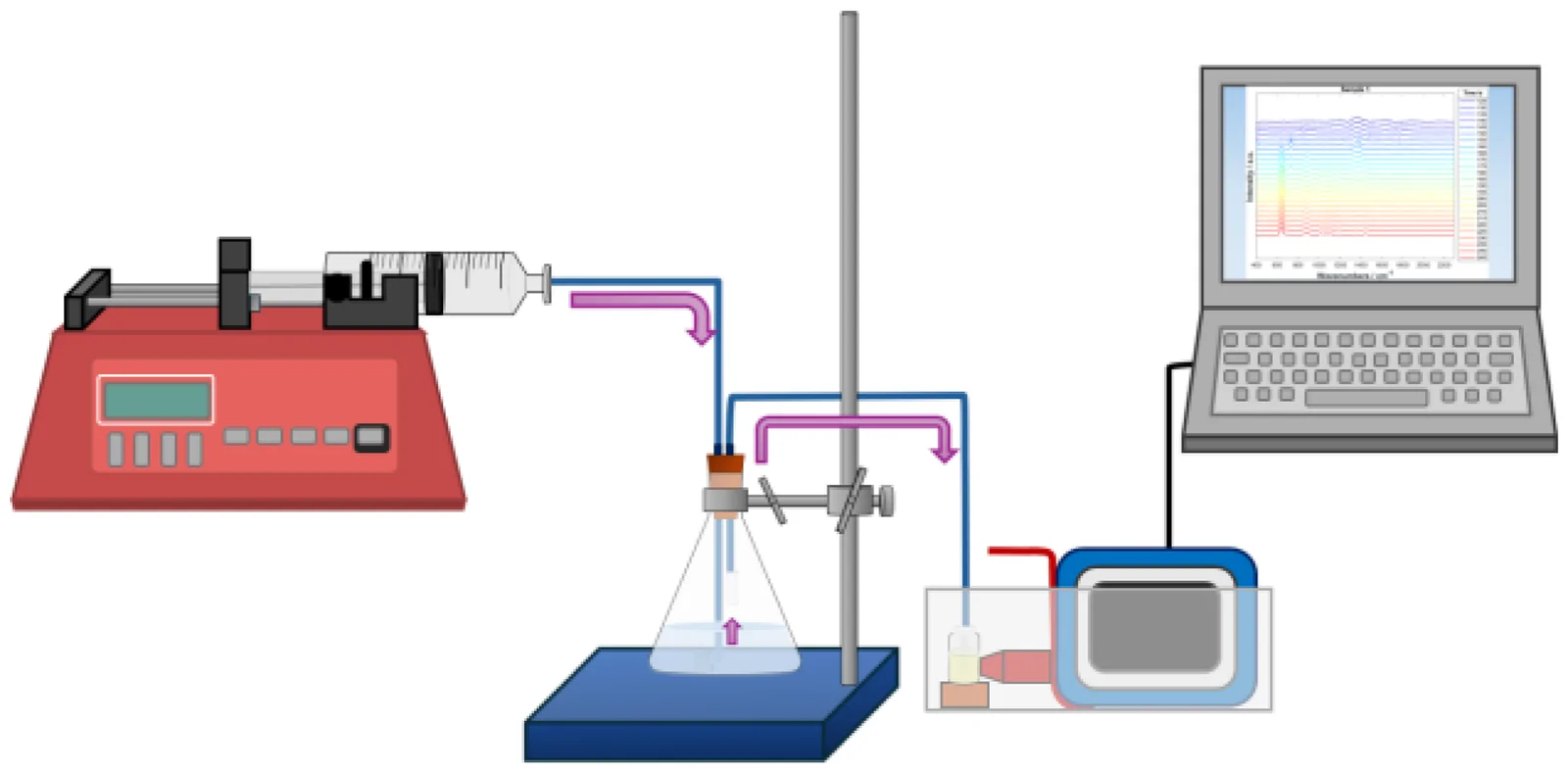

## Introduction

Volatile organic compounds (VOCs) are gaseous molecules characterised by a low molecular weight, high volatility at room temperature and atmospheric pressure, and typically low boiling points.^1–3^ These molecules are produced naturally and synthetically, and their detection offers valuable information across various fields. These include food safety, health and disease, environmental pollution, agriculture, and public safety (such as explosives).^4–9^ The advantage of sensing for VOCs and gaseous molecules is that the methods are usually non-invasive making sample collection less strenuous on participants. Furthermore, samples are normally free of any additional material, such as cellular material from biological samples.^10^ Many VOCs also possess distinctive odour profiles, which can aid in identifying their presence; for example, in body odour, breath analysis, and assessments of food freshness and quality.^11–13^ However, VOCs are usually present in very low concentrations making them difficult to detect.

Current methods for measuring VOCs include gas chromatography-mass spectrometry (GC-MS), electronic nose sensors, selected ion flow tube-MS (SIFT-MS), ultraviolet spectroscopy and infrared spectroscopy.^10,14–20^ Among these, GC-MS and electronic noses are the primary techniques used. GC-MS and other MS-based methods offer high sensitivity and selectivity, with well-established protocols and extensive reference libraries for VOC identification. However, GC-MS typically requires sample pre-concentration techniques such as thermal desorption (TD) or solid-phase microextraction (SPME) for sample measurements. Moreover, GC-MS techniques can be costly, time-intensive, and involve complex sample preparation.^10,21–23^ Finally, samples often need to be transported to the GC-MS meaning VOCs may be lost before analysis.^24^ Electronic noses can be easier to operate than GC-MS, have lower running costs, produce faster results and could be developed to be portable allowing for real-time analysis. On the other hand, they are generally less sensitive and selective than GC-MS – particularly in mixtures of gases – and suffer from interference from moisture in the air.^1,17,23,25^ Hence, new methods need developing that encapsulate the advantages of both electronic noses and MS based techniques.

Surface enhanced Raman scattering (SERS) is another technique which is growing in popularity for the detection of gaseous compounds including VOCs. Typically, solid SERS substrates are produced to perform these experiments and have been used for different applications, including healthcare, microbiology and environmental assessment.^26–29^ The substrates consist of metallic nanostructures (usually gold or silver) manufactured by top-down or bottom-up approaches or by depositing metallic nanoparticles onto a substrate.^30,31^ These methods produce substrates which are portable allowing for real-time analysis. They can also have homogeneous nanostructures and can be sensitive for detecting low concentrations of VOCs. However, due to the high mobility of gaseous molecules, their adsorption onto SERS substrates is often poor. Additionally, many gaseous molecules (such as alcohols) contain functional groups that do not readily bind or adsorb to metallic nanostructures.^15^ As a result, producing effective solid SERS substrates may involve complex fabrication techniques or require specialized expertise to create metal–organic frameworks or porous films that can trap specific VOCs to the metallic surface. Moreover, these substrates can be expensive to manufacture and may vary in reproducibility, sensitivity, and selectivity. Many substrates are tailored for specific target analytes, limiting their versatility across different applications. Consequently, these factors can limit using solid SERS substrates for the detection of VOCs in various fields.

Colloidal metallic nanoparticles are widely used for SERS applications, particularly for the analysis of analytes found in different types of media. Their synthesis methods are established, and they are readily available commercially, making them one of the most accessible options for conducting large-scale experimental measurements. They are also generally cheaper to produce than solid substrates, and improved quantification can be achieved due to signal averaging during measurements.^30,32,33^ Additionally, the continuous Brownian motion of the nanoparticles helps minimize photodegradation of adsorbed analytes and contributes to signal averaging.^32,34^ Nevertheless, there is limited research on using colloidal nanoparticles in solution for the direct detection of gaseous analytes with SERS. Most studies focus on drying nanoparticles onto surfaces or forming thin films for gas-phase SERS analysis. Two methods have previously used for colloidal nanoparticles in solution. One method successfully introduced and measured GC eluates of pyridine into flowing citrate-stabilised silver nanoparticle solutions in a flow cell for online SERS detection.^35,36^ However, as mass spectrometry has become the primary detector for GC-MS, research into this field has been limited.^37,38^ Furthermore, this is also an indirect method of measuring gaseous analytes with nanoparticles in solution. Additionally, microfluidic devices have also been produced to detect gaseous analytes such as 4-aminobenzenethiol and trace vapours of explosives.^39–41^ These devices allow for real-time analysis of gaseous analytes, can be low cost and mass produced and can provide more sensitive results due to the flow of solution allowing for homogenous mixing past the Raman laser. However, microfluidic devices can suffer from memory effects, where residual chemicals linger in the system, requiring cleaning methods like photocatalysis. This could be problematic when trying to analyse strongly adsorbing gaseous molecules such as sulfurous compounds which are difficult to remove without strong chemical reagents. Additionally, microfluidic chips can be complex to produce, and laboratories may not have access to the equipment or expertise to fabricate the devices.^42,43^ Therefore, a simpler SERS method needs developing that is accessible to more laboratories.

The aim of this study was to detect and measure gaseous analytes using SERS with colloidal nanoparticles. For this study, a novel and simple method was developed in which gaseous molecules were bubbled into a stabilised nanoparticles suspension *via* a syringe of air and SERS spectra were recorded using a portable Raman spectrometer. The method was initially tested using 3-mercapto-hexan-1-ol (3MH), as our previous research had found that the thiol aggregated the silver nanoparticles without any additional aggregating agent.^44^ Therefore, this could also happen when the gaseous form of the thiol is bubbled into the nanoparticles. Additionally, 3MH was chosen as the initial interest was developing a method for detecting thiols associated with underarm malodour.^45^ The change in peak height of the C–S peak was monitored and used to optimise the flow rate selected for future measurements. Once optimised, the method was used on two additional analytes: 2-methyl-3-mercapto-pentanol (2M3MP) and indole. The results from this study shows the potential of this technique as an alternative approach for detecting gaseous analytes using colloidal nanoparticles.

## Materials and methods

### Materials

Trisodium citrate, gold(iii) chloride trihydrate (99.999%), sodium hydroxide, hydroxylamine hydrochloride and sodium chloride were purchased from Fisher Scientific (Loughborough, UK). Hydrochloric acid, nitric acid, indole (>99%), and silver nitrate were purchased from Sigma Aldrich Ltd (Dorset, UK). 3-Mercapto-hexan-1-ol (3MH) (96%) was purchased from Alfa Aesar (Heysham, UK). 2-Methyl-3-mercapto-pentan-1-ol (2M3MP) (97%) was purchased from Tokyo Chemical Industry (Oxford, UK).

### Initial testing for bubbling a gaseous analyte into nanoparticles

3MH was chosen as the first analyte to test the bubbling method with because, as shown in our previous research, it causes aggregation of hydroxylamine-stabilised silver nanoparticles (hAgNPs) without the need for an external aggregating agent.^44^ 2.5 mL of 3MH (7 M) was transferred into a 25 mL narrow-necked glass Erlenmeyer flask (Fisher Scientific, Loughborough, UK) and 5 mL of either hAgNPs or citrate-stabilised gold nanoparticles (cAuNPs) were transferred into another 25 mL flask. Silver nanoparticles were synthesised from adopting Leopold and Lendl's method and gold nanoparticles were produced using Lee and Meisel's method.^46,47^ Further details on nanoparticle synthesis and characterisation can be found in SI. Both flasks were sealed using two-hole rubber stoppers (Fisher Scientific, Loughborough, UK). Two lengths of polytetrafluoroethylene (PTFE) tubing (1/16″ inner diameter × 1/8″ outer diameter (OD)) (Avantor, Lutterworth, UK) were inserted into the stopper of the Erlenmeyer flask, fitted with perfluoroalkoxy alkane (PFA) SCAT™ connectors (3.2 mm OD) (Fisher Scientific, Loughborough, UK). One tube was submerged in the 3MH solution and connected to a 20 mL Luer lock syringe *via* a nylon female Luer fitting (Fisher Scientific, Loughborough, UK), which was pre-filled with 20 mL of air. The second tube was positioned in the headspace of the Erlenmeyer flask, with its other end submerged in the nanoparticle solution, allowing the gaseous analyte to be bubbled directly into the colloidal suspension.

20 mL of air was syringed by hand into the 3MH to bubble the gas into the nanoparticles. The nanoparticle solution was left for 30 min before 450 μL was transferred to a vial. The vial was vortexed for 10 s before being measured. Measurements were taken using a DeltaNu Advantage 200A Raman spectrometer (DeltaNu, Laramie, WY, USA) using a 785 nm laser with a typical power on the sample of ∼60 mW. Spectra were acquired over 200 to 2000 cm^−1^ with an acquisition time of 30 s.

### Set up for SERS measurements of gaseous analytes using colloidal nanoparticles

**Fig. 1:**
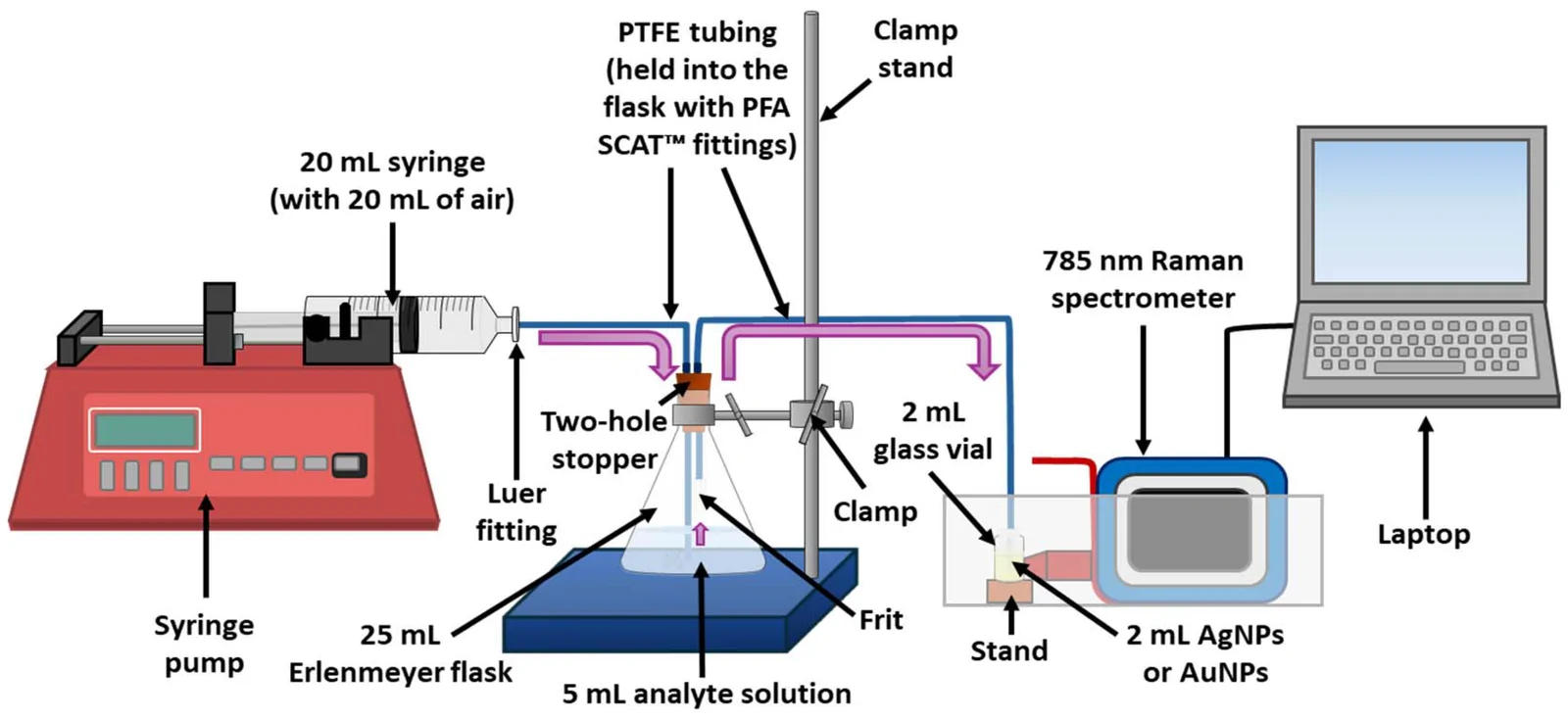
Overview of method used to bubble gaseous analytes into colloidal nanoparticles where the purple arrows indicate the direction of gas flow.

For the measurement of gaseous analytes from a solution or solid, as illustrated in Fig. 1, a 25 mL narrow necked glass Erlenmeyer flask consisting of the sample was sealed with a two-hole rubber stopper. Two lengths of PTFE tubing were inserted through the stopper, secured with PFA SCAT™ fittings to hold the pieces of tubing in place and prevent gas leakage. One tube (270 mm length) was attached to a 20 mL Luer lock syringe using a nylon female Luer fitting. The syringe, filled with 20 mL of air was mounted on a syringe pump to control the flow rate of air through the system. The second tube (300 mm length) was fitted with a PTFE frit (10 μm pore size, 3.2 mm OD) (Fisher Scientific, Loughborough, UK) and positioned in the headspace of the sample in the flask. The other end of the tubing was submerged in a 2 mL glass vial holding 2 mL of silver or gold nanoparticles. The tubing was secured in place using Parafilm®. For each replicate, the tubing between the flask and the vial was replaced to eliminate any residual analyte that could interfere with subsequent measurements.

SERS measurements were performed using a SnRI CBeX handheld Raman spectrometer (Snowy Range, Laramie, Wyoming, USA) equipped with a 785 nm laser and using ∼70 mW of power on the sample. Spectra were collected between 400 and 2300 cm^−1^ with an integration time of 1 s. However, after transferring the spectral data from the spectrometer to the computer, the total spectral acquisition time was 5 s. Measurements were taken in point-and-shoot mode at the glass vial containing the nanoparticles.

### SERS measurements of gaseous analytes using colloidal nanoparticles

To detect gaseous analytes from their liquid counterparts, 5 mL of the analyte was aliquoted into the Erlenmeyer flask. All liquids were prepared to 7 M. For detecting gaseous indole from its solid form, 680 mg of solid indole was placed in the flask and heated on a hotplate at 35 °C throughout the experiment.

The spectral changes as the analyte was bubbled into the nanoparticles were recorded by taking continuous measurements using the Raman spectrometer. For the first 2 min, the nanoparticles were measured without any analyte bubbled into them. After this period, the syringe pump was activated at a set flow rate, causing the analyte to bubble into the nanoparticle solution. Once the syringe was emptied, the pump was switched off and additional spectra were collected for up to 15 min after the pump was switched off.

To facilitate the detection of gaseous indole using gold nanoparticles, 200 μL of NaCl (0.5 M) was pipetted into the nanoparticles in the same timeframe as all the air being pumped out of the syringe. After the pump was switched off, measurements were taken the same way as for the other samples. Six replicates were prepared for this experiment.

### Data analysis

All SERS data were processed using Matlab R2024a (The MathWorks, Natick, MA, USA). For principal component analysis (PCA), spectra were first baseline corrected using asymmetric least squares, smoothed with a Savitzky–Golay filter with a 2^nd^ order polynomial and a window width of 11 points and vector normalised.^48–50^ For measuring the peak heights, individual peaks of interest were chosen from the raw spectra and these peaks were baseline corrected.

To examine how the spectrum changed over time as an analyte was added into the nanoparticles and to evaluate the reproducibility of results across replicates using the same flow rate, the most intense peak in the spectrum of the analyte was chosen and its peak height measured across the different spectra. The rate of change of peak height at 634 cm^−1^ for 3MH was calculated by taking the linear region of the peak height scatter plot for each replicate, calculating the gradient of the linear region and taking the average rate across the three replicates. Standard deviation (SD) and relative standard deviation (RSD) were also calculated to compare different flow rates and find the most reproducible flow rate to use. PCA was carried out as an unsupervised method to reduce the dimensionality of the SERS spectra.^51^ PCA was performed on all the spectra for each replicate and the linear regions of each replicate. The linear regions were determined using the peak height scatter plots for the peak at 634 cm^−1^ and 642 cm^−1^ for 3MH and 2M3MP respectively. Score plots were used to visualise the changes in the spectra over the measurement time and to examine the reproducibility between replicates. Loadings plots were used to examine how the spectra changed over time.

## Results and discussion

### Initial testing for bubbling 3-mercapto-hexan-1-ol (3MH) into nanoparticles and kinetic measurements

To test whether bubbling analytes into colloidal nanoparticles would work for SERS detection of gases, 3MH (vapour pressure of 0.002 kPa at 20 °C) was chosen as the test analyte. This is because the method was originally devised to try and detect gaseous thiols associated with axillary odour with SERS due to failed previous attempts with solid substrates and passive sampling (data not shown). Moreover, 3MH is commercially available and as it is a thiol it contains a sulfur group. It is well known that sulfurous compounds bond to gold and silver nanoparticles and part of the SERS enhancement observed with such compounds is attributed to chemical enhancement *via* metal–sulfur bond formation. Moreover, as seen in our previous research, 3MH in liquid form caused hAgNPs to aggregate at 444 ppm without the need for an aggregating agent.^44^ This made it a suitable candidate to test whether similar aggregation and SERS detection could occur when gaseous 3MH was bubbled into colloidal nanoparticles.

Initially, to assess the feasibility of the method, both hAgNPs and cAuNPs were tested with gaseous 3MH. For this preliminary test, 20 mL of air was manually pumped into the 3MH sample from a syringe instead of *via* a syringe pump. The mixtures were left for 30 min to allow time for the nanoparticles to interact with the gaseous 3MH before being measured. As shown in Fig. S5A, hAgNPs produced an intense SERS spectrum of 3MH. In contrast, a spectrum was generated with cAuNPs but the peaks for 3MH were less intense. When comparing the spectrum collected by bubbling gaseous 3MH into hAgNPs with the spectrum from mixing liquid 3MH into hAgNPs and aggregating with NaCl, the same characteristic peaks were observed (Fig. S5B); although the spectrum is not as intense as the SERS spectrum of liquid 3MH at 3 ppm, which could indicate that the concentration of gaseous 3MH in the nanoparticle solution is lower than 3 ppm (Fig. S5C). Nevertheless, this showed that SERS measurements can be obtained from bubbling gaseous 3MH into hAgNPs.

**Fig. 2:**
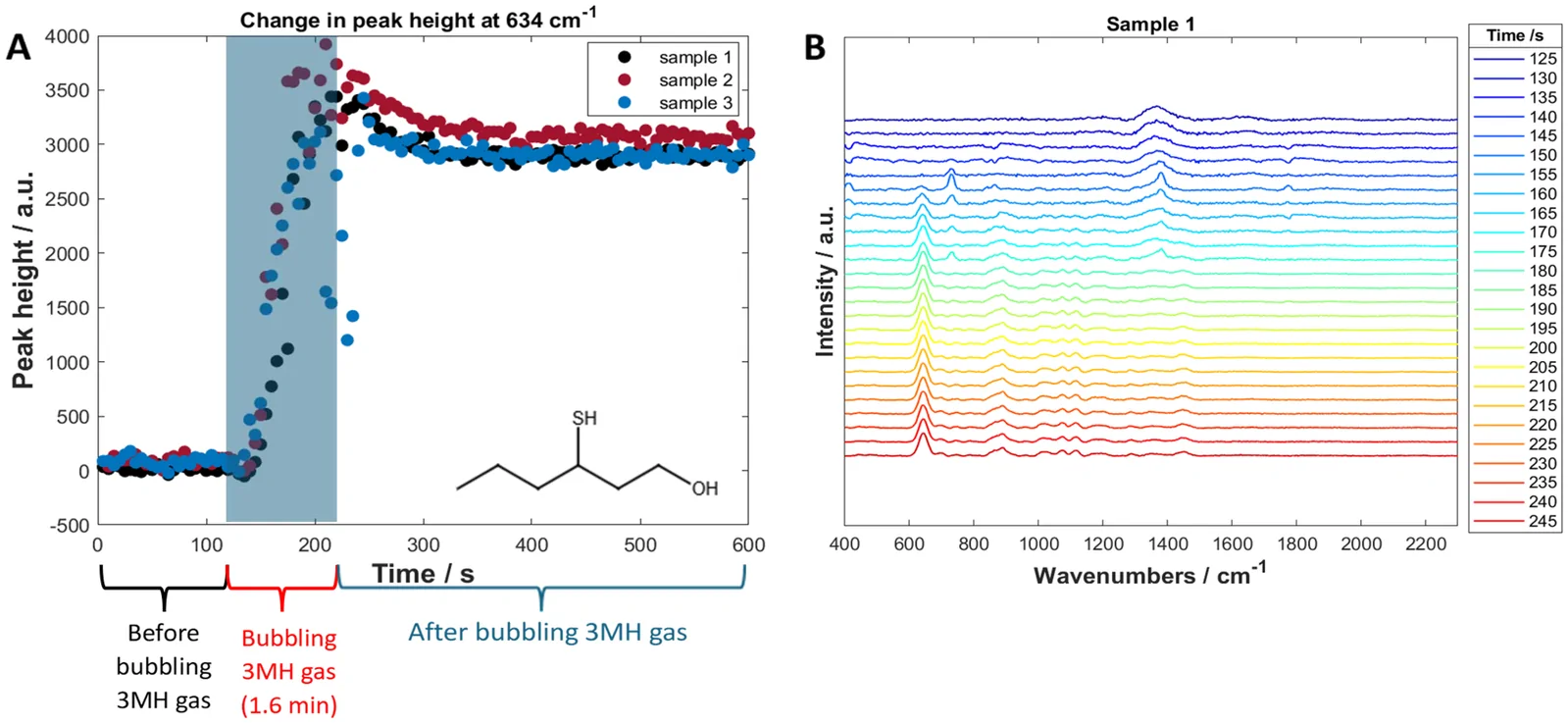
(A) Kinetic graph of changes in peak height at 634 cm^−1^ across the duration of the bubbling experiment at a flow rate of 12.5 mL min^−1^; for 3 repeats. The region highlighted in blue represents the timeframe when 3MH was bubbled into hAgNPs. (B) Spectra of 3MH changing from the start (blue) of the bubbling period to the timepoint at which the spectra no longer changes (red) for the first replicate. The kinetic region was found from the linear region of the change in peak height at 634 cm^−1^. The spectra have been baseline corrected, smoothed and vector normalised.

In addition to being able to detect gaseous analytes, the bubbling method was tested to see if kinetic measurements could be taken by collecting spectra whilst the thiol was being bubbled into hAgNPs. For the first 2 min of measurements, no analyte was introduced into the nanoparticles. Then, 3MH was bubbled into hAgNPs at a flow rate of 12.5 mL min^−1^. To pump all the air from the syringe took 1.6 min to complete. Spectra were then measured for a further 6 min to monitor any further changes as the analyte interacted with the nanoparticles. This was done with three replicate samples. As 3MH was bubbled into the colloidal solution, the analyte began to interact with the nanoparticles and likely started to form bonds to the nanoparticle surfaces. This interaction was evidenced by the appearance of a peak at 634 cm^−1^ (the *gauche v*(C–S) band) in the spectrum for 3MH as it was bubbled into hAgNPs (Fig. 2B and Fig. S6). Since the sulfur atom in the thiol forms a bond to the nanoparticle surface, vibrational modes associated with the C–S bond are expected to be more intense and appear earlier in the kinetic profile than other spectral features. To further examine this, the peak height at 634 cm^−1^ was measured across the spectra collected during the bubbling experiment. As more 3MH was bubbled into hAgNPs, the peak height increased (Fig. 2A). Once the syringe was emptied which caused the bubbling to cease, the peak height at 634 cm^−1^ appeared to increase slightly then plateaued and remained constant for the rest of the measurements. This could be because the nanoparticle surface is saturated with chemically bound 3MH. Therefore, the peak height does not change once all nanoparticle surface is occupied. These findings demonstrate that the bubbling method can be used for kinetic measurements.

**Fig. 3:**
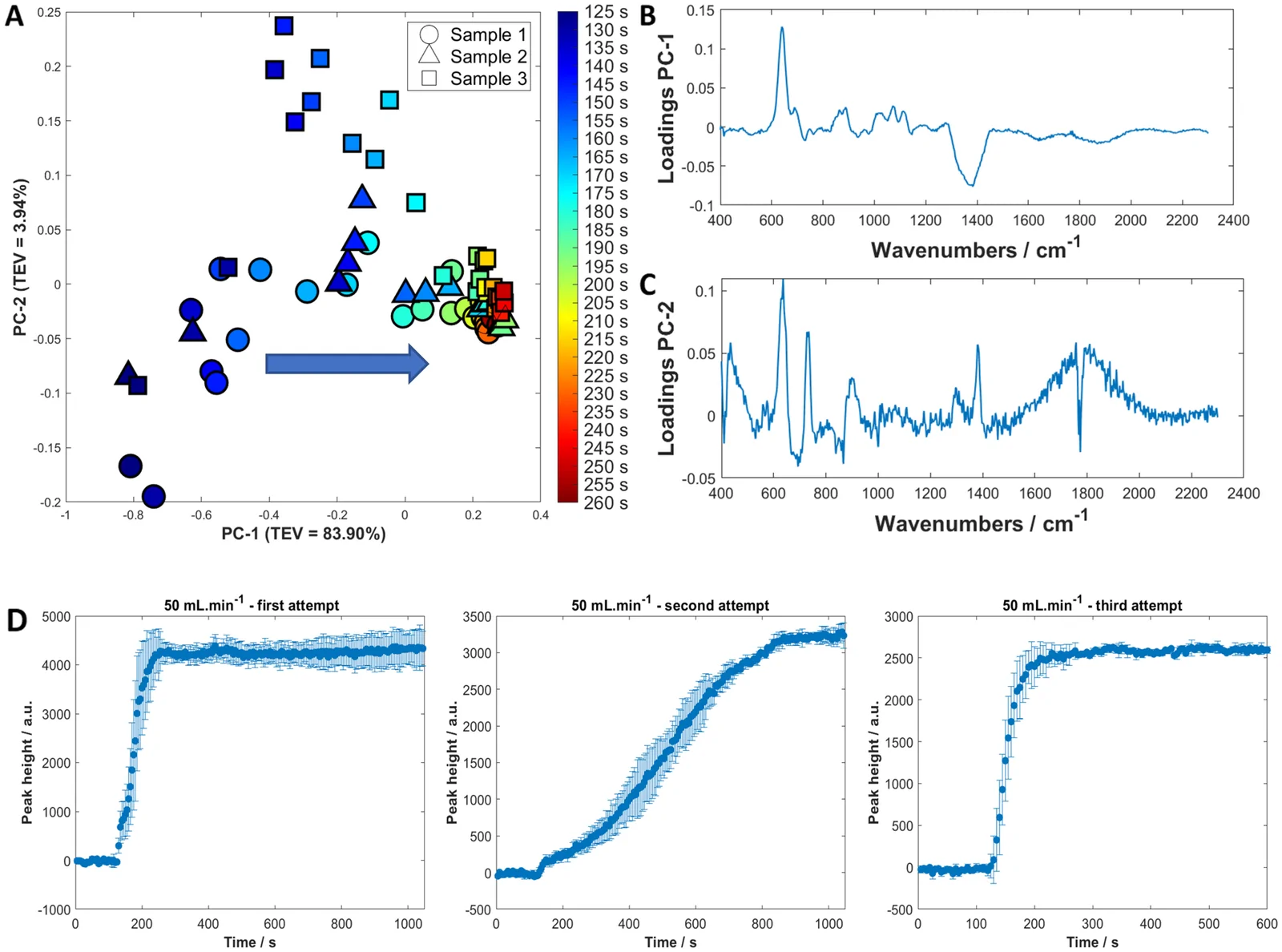
(A) PCA scores plot of the spectra changing between 125 s (blue) and 280 s (red) during the kinetic region of the bubbling experiment at 50 mL min^−1^. The arrow indicates direction of decreasing concentration and TEV is the total explained variance. (B) Loadings plot of PC-1 for the linear region in the kinetic experiment at 50 mL min^−1^. (C) Loadings plot of PC-2 for the linear region in the kinetic experiment at 50 mL min^−1^. (D) Average (*n* = 3) kinetic graphs of change in peak height at 634 cm^−1^ across the bubbling experiment for first attempt, second attempt and third attempt at a flow rate of 50 mL min^−1^. Each attempt was taken on a different day. Error bars represent the standard deviations from the three repeat measurements within each attempt. Data for figures (A–C) have been baseline corrected, smoothed and vector normalised.

Following on from these first tests, to improve the reproducibility of the results obtained, the gas flow rate was optimised. For this, eight different flow rates between 2 and 50 mL min^−1^ were tested. To evaluate the flow rates, the peak height at 634 cm^−1^ was calculated for each spectrum and depicted in a kinetic graph (Fig. S7). PCA models were also produced from all the spectra for each replicate at each timepoint and from the spectra corresponding to the linear region of the 634 cm^−1^ peak height scatter graphs (Fig. 3A–C and Fig. S9–S16). Full details and results of the flow rate optimisation are provided in the SI. Overall, across all the PCA scores and loadings plots, a trend was seen in PC-1 from negative to positive score values with increasing time (Fig. 3A). The loadings plot for PC-1 confirmed that the variation in the spectra over time was driven by the appearance of peaks associated to 3MH (Fig. 3B). Additionally, 50 mL min^−1^ showed the best results across three repeat experiments of flow rate optimisation (Fig. 3D). This could be due to the faster bubbling and slower aggregation impacting the mixing and interactions between the nanoparticles and 3MH and making the results more reproducible (further discussion on these points can be found in the SI) (Fig. S5B and Table S1). However, the results are different between attempts due to differences in tubing lengths used as they were being optimised in the other two attempts. This could also be why attempt 2 showed differences in reaching saturation point in comparison to the other two attempts as the tubing length was longer (*ca*. 40 cm compared to ∼30 cm) between the vial containing nanoparticles and the conical flask containing 3MH, and may lead to more binding of the 3MH during the analysis. However, despite this the replicates for all three attempts were good and therefore 50 mL min^−1^ was used for all further testing.

### Testing 2-methyl-3-mercapto-pentan-1-ol (2M3MP) and indole with the bubbling system at 50 mL min^−1^

To determine and understand what physicochemical properties of molecules could influence if an analyte can be measured with this SERS bubbling system, two other VOCs with different molecular characteristics were tested. These were: 2-methyl-3-mercapto-pentan-1-ol (2M3MP) and indole. Both analytes were investigated at the same molarity as 3MH. These compounds were selected as they contain elements such as sulfur or nitrogen, which are known to interact with Au or Ag nanoparticles. Furthermore, these analytes may also cause nanoparticle aggregation in the same way as 3MH.

**Fig. 4:**
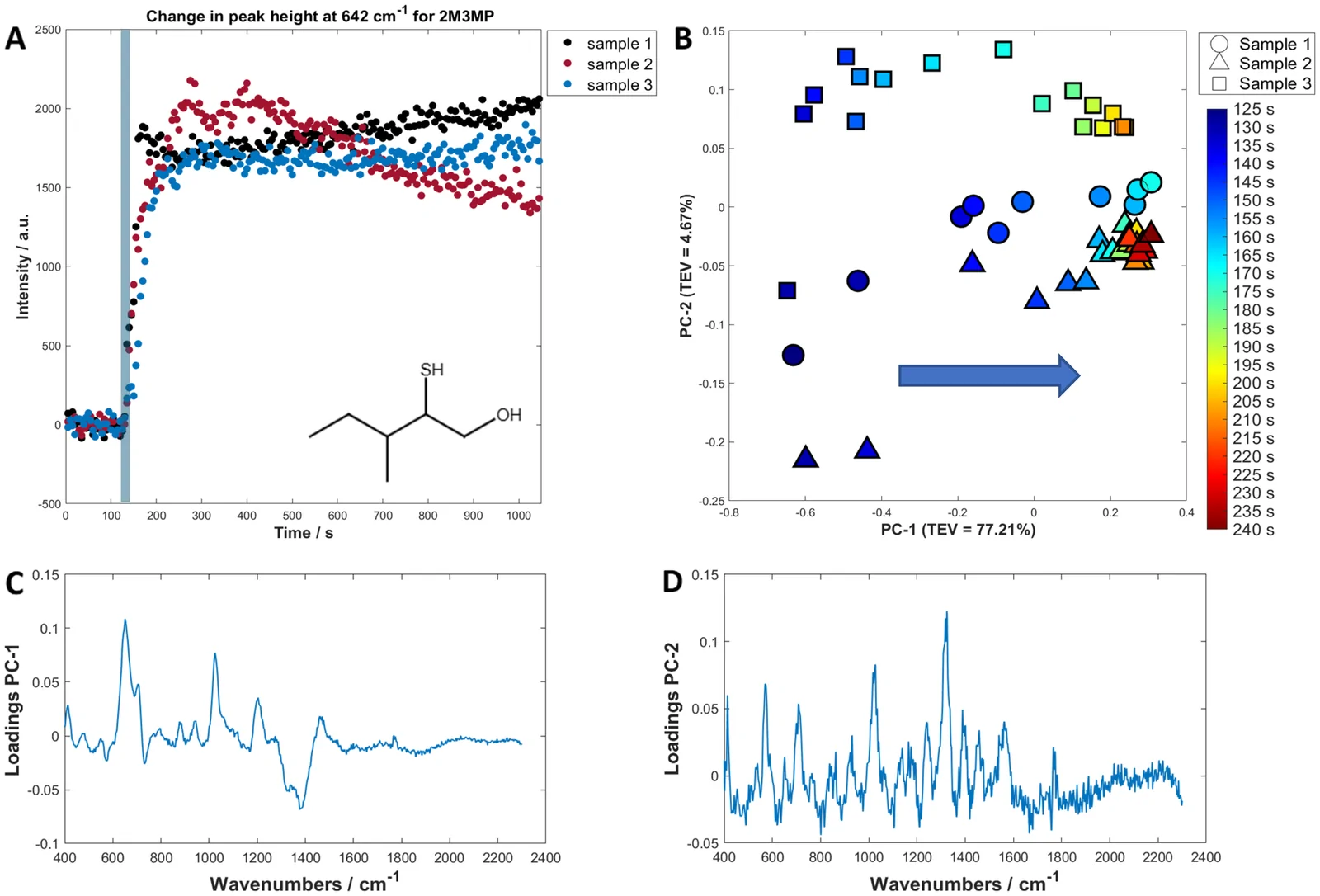
(A) Kinetic graph of changes in peak height at 642 cm^−1^ for 2M3MP across the duration of the bubbling experiment at 50 mL min^−1^ for the three replicate measurements. The region highlighted in blue represents the timeframe when 2M3MP was bubbled into hAgNPs. (B) A PCA scores plot of the spectra changing between 125 s (blue) and 240 s (red) during the kinetic regions for the different replicates of the bubbling experiment for 2M3MP at 50 mL min^−1^. The arrow indicates direction of decreasing concentration and TEV is the total explained variance. (C) Loadings plot of PC-1 for the linear region in the kinetic experiment at 50 mL min^−1^. (D) Loadings plot of PC-2 for the linear region in the kinetic experiment at 50 mL min^−1^. Data for figures (B–D) have been baseline corrected, smoothed and vector normalised.

Initially, 2M3MP (a thiol compound) (vapour pressure currently not defined) was measured to assess the system's performance with another sulfur-containing VOC. 2M3MP is also produced in underarm odour and in *allium* plant species like onions, leeks and chives.^52^ 2M3MP as a liquid has previously been successfully detected with SERS using hAgNPs.^44^ Therefore, these were used again to try to detect gaseous 2M3MP. Similar to 3MH, the change in peak height of the *gauche v*(C–S) band at 642 cm^−1^ was measured across the spectra and plotted for all three replicates (Fig. 4A). As with 3MH, an increase in peak height at 642 cm^−1^ was observed as 2M3MP was bubbled into hAgNPs. Moreover, when PCA was used to look at all the spectra and the spectra in the linear regions of the scatter graphs, the trend in PC-1 from negative to positive values looked to be due to the appearance of peaks associated to 2M3MP (Fig. 4B and Fig. S18). Compared to 3MH, 2M3MP showed lower reproducibility with PC-2 TEV value of 4.67% for the linear region. This could be due to differences in how 2M3MP interacts with hAgNPs. When liquid 2M3MP was mixed with hAgNPs without an aggregating reagent, a SERS spectrum could not be generated. This could be because 2M3MP has more side chains than 3MH, making it a bulkier molecule. Hence it may be that it is harder to form Ag–S bonds with 2M3MP and the spectra are weaker in intensity. Alternatively, for the liquid measurements, the concentration of 2M3MP used may have been too low. Overall, this demonstrates that this method can be used for sulfurous molecules. Therefore, SERS spectra of 3MH and 2M3MP can be measured due to their shared physiochemical properties.

To determine which molecular properties are important for SERS analysis using this system, indole was also analysed. Indole (vapour pressure of 0.0016 kPa at 25 °C) is a VOC produced by *Escherichia coli* from the enzymatic conversion of tryptophan to indole by tryptophanase.^53–55^ It can be used to differentiate between bacteria and identify bacterial infections.^56^ Indole consists of pyrrole ring which is a nitrogen-containing functional group. Nitrogen is also known to associate with metallic nanoparticles. This leads to chemical enhancement with SERS.

As indole is not water-soluble, methanol was used to dissolve indole due to its minimal interference with indole's Raman spectrum as the Raman spectrum of methanol only has a few peaks present. When comparing the spectra of cAuNPs and hAgNPs with liquid indole, cAuNPs were selected for further measurements since they provided stronger spectral enhancement of indole (Fig. S19A). However, when indole was added to both types of nanoparticles, no aggregation could be seen visually. When gaseous indole was bubbled into cAuNPs from the liquid indole, the change in peak height at 760 cm^−1^ was analysed (out-of-phase pyrrole ring breathing vibration).^56^ As seen in Fig. S19B, no peak at 760 cm^−1^ could be observed before, during or after the bubbling process. These results, suggest that for an analyte to be detected in its gaseous form, it must both induce nanoparticle aggregation and form a bond with the nanoparticles. One way to cause nanoparticle aggregation is to adjust the pH of the solution inducing changes in the surface charge of the nanoparticles. As indole is not as basic as other amines and nitrogen-containing compounds (such as ammonia), it does not cause nanoparticles aggregation. Basic environments cause nanoparticle aggregation due to cations present in the solution shielding the negative surface charge of the nanoparticles. This reduces repulsion and promotes nanoparticle aggregation. This is likely why indole could not be detected when it was bubbled into the nanoparticles. In comparison, the thiols are mildly acidic. In acidic environments, the reducing agent surrounding the metallic core is protonated, lowering repulsion between the nanoparticles, causing aggregation. Simultaneously, the deprotonated sulfur on the thiol bonds to the metal and is enhanced by chemical enhancement and from being present in the hotspots caused by aggregation. This is likely why 3MH and 2M3MP cause the nanoparticles to aggregate and allowing for detection through this method.

### Aggregating nanoparticles for the detection of indole

**Fig. 5:**
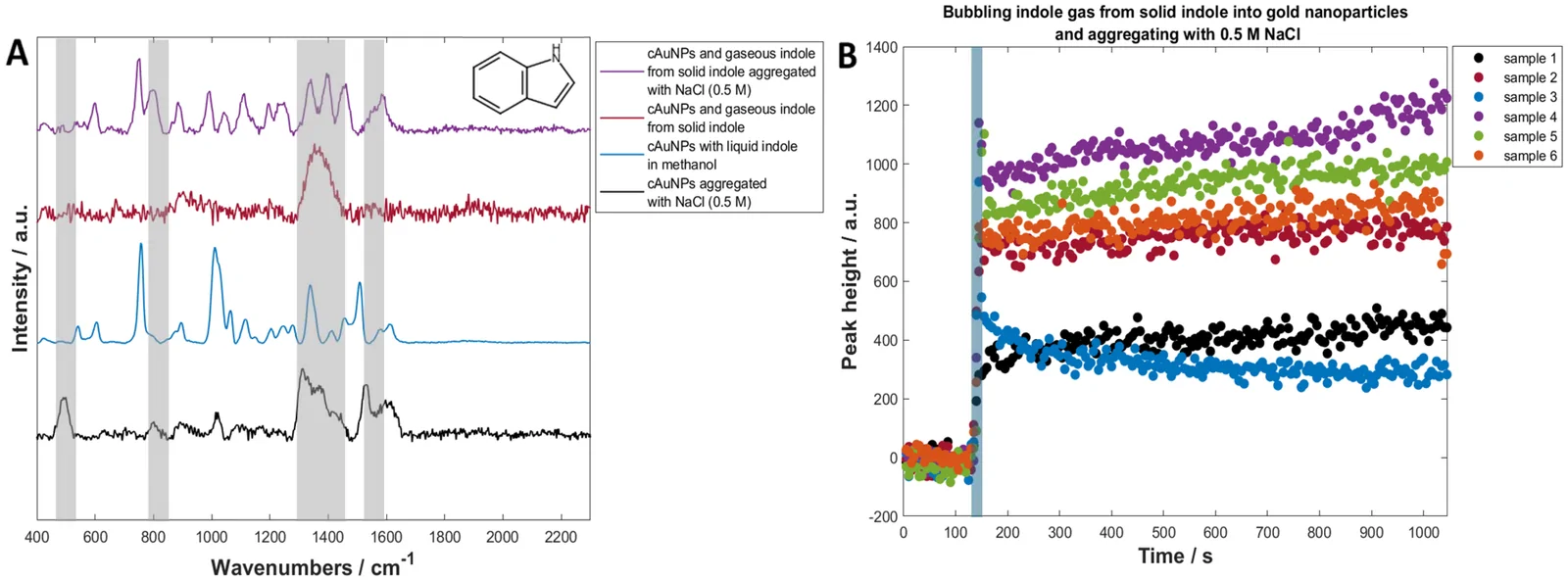
(A) SERS spectra of cAuNPs aggregated with 0.5 M NaCl (black), cAuNPs with indole dissolved in (liquid) methanol (blue), cAuNPs with gaseous indole produced from solid indole with no aggregating reagent (red) and cAuNPs with gaseous indole produced from solid indole with 0.5 M NaCl (purple). Peaks highlighted in grey represent peaks found in the spectrum of cAuNPs aggregated with 0.5 M NaCl. The spectra have been baseline corrected, smoothed and vector normalised. (B) Kinetic graph of changes in peak height at 760 cm^−1^ for indole across the duration of the bubbling experiment at 50 mL min^−1^ for the six replicate measurements. The region highlighted in blue represents the timeframe when indole was bubbled into cAuNPs.

To try and improve the detection of gaseous indole, solid indole was heated at 35 °C to produce the vapour. At the same time as the indole was being bubbled in, 200 μL of 0.5 M NaCl (as an aggregating agent) was manually pipetted into the nanoparticles. This was to try and cause indole to bond to the nanoparticles or for indole to be caught in hotspot regions formed during aggregation. When comparing spectra with and without NaCl, peaks are present in the spectrum when an aggregating agent is used (Fig. 5A). The spectrum appears to show a mix of peaks from the aggregated nanoparticles and indole. Notably, the peak at 760 cm^−1^ which is found in the spectrum of indole. Therefore, the change in peak height at 760 cm^−1^ was measured when adding NaCl as indole was bubbled into nanoparticles. Six measurements were taken to examine the reproducibility of the results. As seen in Fig. 5B, as indole was bubbled and NaCl was pipetted into the nanoparticles, the peak height at 760 cm^−1^ increased and then plateaued. This is also seen with the results for 3MH and 2M3MP. However, clear differences in reproducibility were identified between the six replicate measurements. This was primarily due to the human error of trying to pipette NaCl for the same duration as bubbling indole into cAuNPs. This caused variations in the time of aggregation and the resulting peak heights for the spectrum of indole. To improve reproducibility, a more automated approach of adding the aggregating agent would be needed. Nevertheless, the results demonstrate how an aggregating agent could be used for detecting analytes which do not naturally induce nanoparticle aggregation and may not bond to the nanoparticle surface.

## Limitations and future work

While the method has demonstrated the detection of gaseous analytes with colloidal nanoparticles for SERS, the current setup is a proof of concept system and requires engineering improvements to provide more reproducible and reliable results. Especially before using the setup for real-time applications such as studying the headspace of bacteria or monitoring VOCs produced from breath. So far, only the flow rate has been optimised, meaning there are other parameters that could also be optimised to improve the results. These include the volume of nanoparticles, the volume ratio of nanoparticles to analyte, the volume of gas bubbled into the nanoparticles and the lengths of tubing used. Environmental factors such as temperature and humidity also vary daily and seasonally so should be controlled, as these factors will affect the amount of gaseous analyte produced.

The system needs additional design features to prevent gas leakage. Currently, the tubing is being held in place with parafilm, which is not a reliable seal for the vial of nanoparticles and is likely allowing some gaseous analyte to escape. Therefore, stoppers are needed that can also hold the tubing in place. Further containment of the whole system would also prevent any more gas escaping and improve the reproducibility of the results. Furthermore, the tubing between the flask and vial must be replaced after each measurement due to chemical memory effects as the gaseous metabolites bind to the tubing during the analysis. Cleaning methods for the tubing should be investigated to reduce the frequency of replacements.

For measuring VOCs that do not cause the nanoparticle aggregation, an aggregating agent is needed, as demonstrated in these studies for indole. As currently the aggregating agent is manually pipetted into the nanoparticles during bubbling, this reduces the reproducibility of the results. Therefore, automation and optimisation of when to add the aggregating agent to the nanoparticles is necessary. Testing this method with an analyte such as acetic acid is also needed to confirm if aggregation-induced hotspots and clustering of the nanoparticles helps to trap and measure the gaseous analytes. Moreover, as with looking at liquids with colloidal nanoparticles, the type and concentration of aggregating agent needs optimising for each analyte of interest.

Other methods that could be examined to try and improve detection of VOCs using colloidal nanoparticles include using SERS tags bound to the nanoparticles. The tag would need to bind to the analyte of interest to bring it closer to the nanoparticles, though this would make the method targeted for specific analytes. The SERS tag would have to change depending on the target analyte. As well as automation for adding the aggregating agent, incorporating computational aid, such as automating the start and stop of bubbling, could improve the ease of use and reproducibility of the results.

Finally, the method should be tested with lower concentrations of analyte and limits of detection should be calculated for the analytes studied in this research; although currently this is beyond the current experimental rig. Moreover, to determine the system's suitability for more complex matrices, the method should also be tested with multiple gaseous analytes simultaneously to evaluate its potential for multiplex detection.

## Conclusion

Research into performing SERS for the detection and assessment of gaseous molecules, especially VOCs, has largely focused on developing solid substrates. However, there are limitations for implementing solid SERS substrates into wider applications and fields. Although colloidal nanoparticles in solution also present challenges for real life applications, they can be cheaper and easier to batch synthesise compared to solid substrates.^33^ Hence, this research explored developing a simple method for the measurement of gaseous analytes using colloidal nanoparticles in solution.

For this, a basic setup was designed and used in which 20 mL of air was pumped from a syringe into a conical flask holding the analyte using a syringe pump. This caused the analyte to bubble into a vial containing the metallic nanoparticle suspension. Spectra were then recorded before, during and after the bubbling process using a portable Raman spectrometer. Using this approach, gaseous 3MH was successfully measured with hAgNPs. It is assumed that this is because 3MH induced nanoparticle aggregation and bonded to the nanoparticles.

Subsequently, 3MH was used to optimise the gas flow rate into the nanoparticle solution. Eight flow rates at 2, 4, 5, 6, 8, 10, 20 and 50 mL min^−1^ were tested. The peak height at 634 cm^−1^ was tracked across the timeframe of the experiments. Ultimately, 50 mL min^−1^ was found to be the most reproducible flow rate and was used in subsequent experiments.

To further evaluate this optimised method and assess its applicability to other gaseous molecules, two other analytes were tested. The analytes chosen were: 2M3MP and indole. The results pointed to two key factors for successful detection using this setup. Firstly, the analyte must cause nanoparticle aggregation in some manner to generate hotspot regions. This can be achieved by altering the pH of the nanoparticle solution. Secondly, the analyte should bind or remain in close proximity to the nanoparticle surface to help prevent the escape of gaseous analytes from the mixture. Therefore, gaseous 2M3MP was also detectable. However, for analytes which do not elicit self-aggregation such as indole, an aggregating agent can be added whilst the analyte is bubbled into the nanoparticles. This needed to be done with indole being measured with cAuNPs. Although the method of using an aggregating agent requires further refinement by using a syringe pump to improve reproducibility.

In conclusion, this SERS-based approach using colloidal nanoparticles in solution shows good potential for real-time detection of VOCs across various sample types.

## Author contributions

A. C. contributed towards conceptualisation, experimental design, investigation, data analysis, data interpretation and writing the original draft. N. G. contributed towards experimental design. S. M. contributed towards experimental design, and supervision. R. G. contributed towards conceptualisation, experimental design, data interpretation and supervision. All authors contributed towards reviewing and editing the writing.

## Conflicts of interest

The authors declare no competing financial interest.

## Supplementary Material

AN-151-D6AN00673F-s001

## Data Availability

Data processing algorithms are available *via*: https://github.com/Biospec/. Supplementary information (SI) is available. Supplementary Information (SI) includes: additional experimental methods for nanoparticle synthesis and characterisation and flow rate optimisation, results from nanoparticle characterisation and flow rate optimisation, Spectra of gaseous 3MH measured with either cAuNPs or hAgNPs, Spectra over time of other replicates of bubbling 3MH into hAgNPs, PCA results for bubbling 2M3MP and initial results from attempting to measure gaseous indole using cAuNPs. See DOI: https://doi.org/10.1039/d6an00673f.
