# Supplementary material for: Analysing volatile organic compounds (VOCs) with SERS by bubbling into colloidal nanoparticles
Source: Analyst. 2026 Aug 17;151(20):5900–10. doi: 10.1039/d6an00673f (PMC13479307; doi:10.1039/d6an00673f)
Supplement: Supplementary file 1 [file AN-151-D6AN00673F-s001.pdf]

Supplementary Information (SI) for Analyst.
This journal is © The Royal Society of Chemistry 2026

Supporting Information

Analysing volatile organic compounds (VOCs) with SERS by bubbling into colloidal nanoparticles

Amy Colleran,[a] Nigel Gotts,[a] Stephanie Murray[b] and Royston Goodacre[a*]

[a] Centre for Metabolomics Research, Department of Biochemistry, Cell and Systems Biology, Institute of Systems, Molecular and Integrative Biology, University of Liverpool, Crown St, Liverpool, L69 7ZB, UK;

[b] Unilever Research and Development, Port Sunlight, Bebington, CH63 3JW, UK

*Correspondence to: roy.goodacre@liverpool.ac.uk

Table of Contents

Methods S2

Gold and Silver Nanoparticle Synthesis S2

Nanoparticle Characterisation S2

Flow rate testing S3

Nanoparticle Characterisation S3

Figure S1: UV-Vis absorption spectra of citrate-stabilised gold nanoparticles and hydroxylamine stabilised silver nanoparticles S3

Figure S2: SEM images of citrate-stabilised gold nanoparticles and hydroxylamine stabilised silver nanoparticles S4

Figure S3: Histograms of the average hydrodynamic diameters (n = 5) of citrate-stabilised gold nanoparticles and hydroxylamine stabilised silver nanoparticles S5

Figure S4: Average zeta potentials (n = 5) of citrate-stabilised gold nanoparticles and hydroxylamine stabilised silver nanoparticles S6

Figure S5: Average zeta potentials (n = 5) of citrate-stabilised gold nanoparticles and hydroxylamine stabilised silver nanoparticles S7

Figure S6: Spectra of 3MH changing from the start (blue) of the bubbling period to the timepoint at which the spectra no longer changes (red) for the second replicate and third replicate; with the first replicate shown in Figure 2. .... S8

Optimising flow rate S8

Figure S7: Kinetic graph of changes in peak height at 634 cm^{-1} across the duration of the bubbling experiment at different flow rates between 2 and 50 $mL.min^{-1}$. S10

Figure S8: Average kinetic graphs of changes in peak height at 634 cm^{-1} and error plots of average rate of change across the duration of the bubbling experiment at different flow rates S11

Table S1: Average rate of change in peak height at 634 cm^{-1} calculated from the linear region of the kinetic graph of each replicate (n = 3), standard deviations and relative standard deviations (RSD) at the different flow rates tested.. .... S12

Figures S9 to S16: PCA scores and PC-1 and PC-2 loadings plots for the kinetic regions and all spectra measured at each timepoint for each replicate for the different flow rates tested. S13-S20

Figure S17: Raman spectrum of PTFE tubing used for transporting the gaseous analyte into the vial of metallic nanoparticles. S20

Figure S18: PCA scores and PC-1 and PC-2 loadings plots for the kinetic regions and all spectra measured at each timepoint for each replicate for 2M3MP at 50 mL.min^{-1}. S21

Figure S19: Raman spectrum of indole in methanol, SERS spectra of indole in methanol produced using hAgNPs and cAuNPs and kinetic graph of changes in peak height at 760 cm-1 for indole across the duration of the bubbling experiment at 50 mL.min-1 for the three replicate measurements.. .... S21

References.. S21

Methods

Gold and Silver Nanoparticle Synthesis

All glassware used for the synthesis of gold and silver nanoparticles was cleaned with aqua regia (HNO_3 : HCl, 1:3, v/v) to remove any residual metals, followed by a thorough rinsing with deionised water. The glassware was dried and sterilised through autoclaving before use.

Synthesis of hydroxylamine-stabilised silver nanoparticles (hAgNPs)

Hydroxylamine-stabilised silver nanoparticles were synthesised by adopting the Leopold and Lendl method.[1] 20 mL of sodium hydroxide solution (30 mM) was dissolved into 158 mL of deionised water, under continuous stirring, followed by 2 mL hydroxylamine hydrochloride solution (150 mM). To this solution, 20 mL of diluted silver nitrate solution (10 mM) was slowly added dropwise to the sodium hydroxide/water solution. The solution was left stirring for 15 min. A change in colour of the solution was observed from colourless to an orange/grey which indicated successful nanoparticle formation.

Synthesis of citrate-stabilised gold nanoparticles (cAuNPs)

Citrate-stabilised gold nanoparticles were synthesised using the Lee and Meisel method.[2] Chloroauric acid (0.24 g) was dissolved into 500 mL of deionised water and brought to the boil, with continuous stirring. Trisodium citrate (1.0 g) was dissolved in 100 mL deionised water and 50 mL of trisodium citrate solution was then slowly added dropwise to the chloroauric acid/water solution. The solution was left to boil for 1 h and left to return to room temperature. A change in colour of the solution was observed from yellow to a dark red/purple indicating successful Au nanoparticle formation.

Nanoparticle Characterisation

UV-Vis spectroscopy was used to determine the surface plasmon resonance band λ_{max} of the different nanoparticles. 2 mL nanoparticle suspension was pipetted into a quartz pipette (path length= 10 mm) and inserted into the sample holder of a Jenway 7200 scanning spectrophotometer (Cole-Parmer, Staffordshire, UK). Absorbance spectra were obtained over a wavelength range of 300-800 nm. Hydroxylamine-stabilised silver nanoparticles (hAgNPs) were diluted in a 1:4 ratio in deionised water and citrate-stabilised gold nanoparticles (cAuNPs) were diluted in a 1:3 ratio in deionised water. Scanning electron microscopy (SEM) was used examine the morphology of the nanoparticles. The nanoparticles were deposited on to Si wafers and imaged using a Hitachi S-4800 SEM. The hydrodynamic diameter and zeta-potential of the nanoparticles were measured using the Malvern Zetasizer Nano ZS (Malvern Panalytical, Malvern, UK) fitted with a 532 nm laser.

Flow rate testing

The flow rate was optimised using 3-mercapto-hexan-1-ol (3MH) as the analyte of interest. The flow rates tested were 2, 4, 5, 6, 8, 10, 20 and 50 mL.min^{-1}. This meant the bubbling time and time it took to empty the 20 mL syringe of air was 10, 5, 4, 3.33, 2.5, 2, 1 and 0.4 min respectively. Three replicates were measured for each sample.

Nanoparticle Characterisation

Citrate-stabilised gold nanoparticles (cAuNPs) and hydroxylamine-stabilised silver nanoparticles showed absorption maxima at 537 nm and between 414 and 424 nm with UV-Vis spectroscopy. The wavelength is in agreement with the literature for cAuNPs and shorter than previously discussed for hAgNPs.[2, 3]

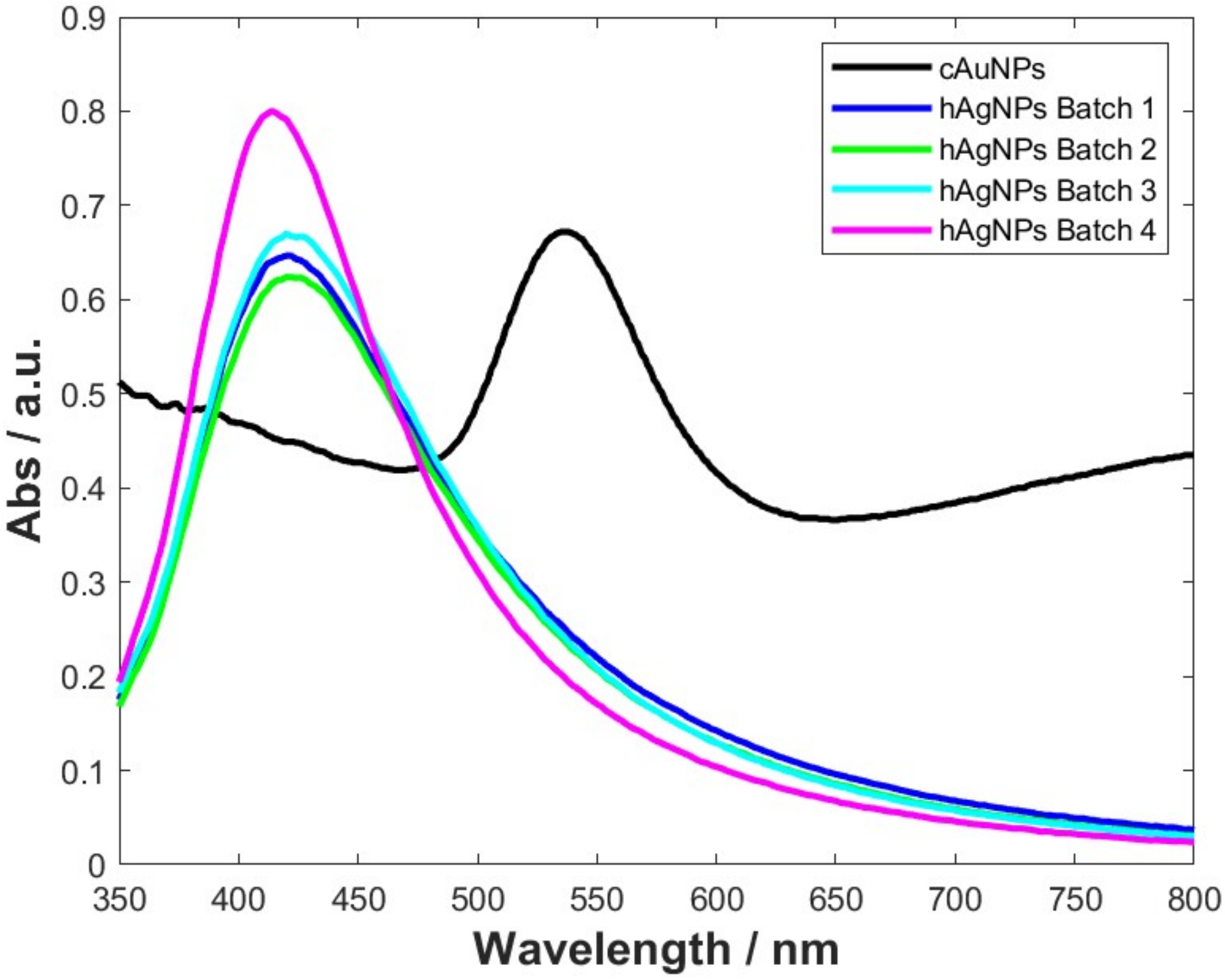

Figure S1: UV-Visible absorption spectra of citrate-stabilised gold nanoparticles (cAuNPs) and hydroxylamine-stabilised silver nanoparticles (hAgNPs).

To characterise the nanoparticles further, hydrodynamic diameter, zeta-potential and size distribution of the nanoparticles was measured using dynamic light scattering (DLS). cAuNPs have a hydrodynamic diameter of 50.27 ± 0.19 nm and hAgNPs batches 1 to 4 appeared to have diameters between 65.48 ± 0.52 nm and 76.03 ± 0.56 nm (**Figure S3**). hAgNPs batch 4 are smaller in diameter than batches 1 to 3. Batches 1 and 2 was used for all measurements discussed in this research. All the nanoparticles had polydispersity indexes were greater than 0.19 indicating there was some polydispersity present. All batches and types of nanoparticles showed a zeta-potential value between -34 mV and -38 mV. Therefore, all nanoparticles produced were stable in solution.[4] However, cAuNPs have a higher RSD

value in comparison to the hAgNPs (6.21%) (**Figure S4**). This indicates that some of the nanoparticles in solution may not be as stable.

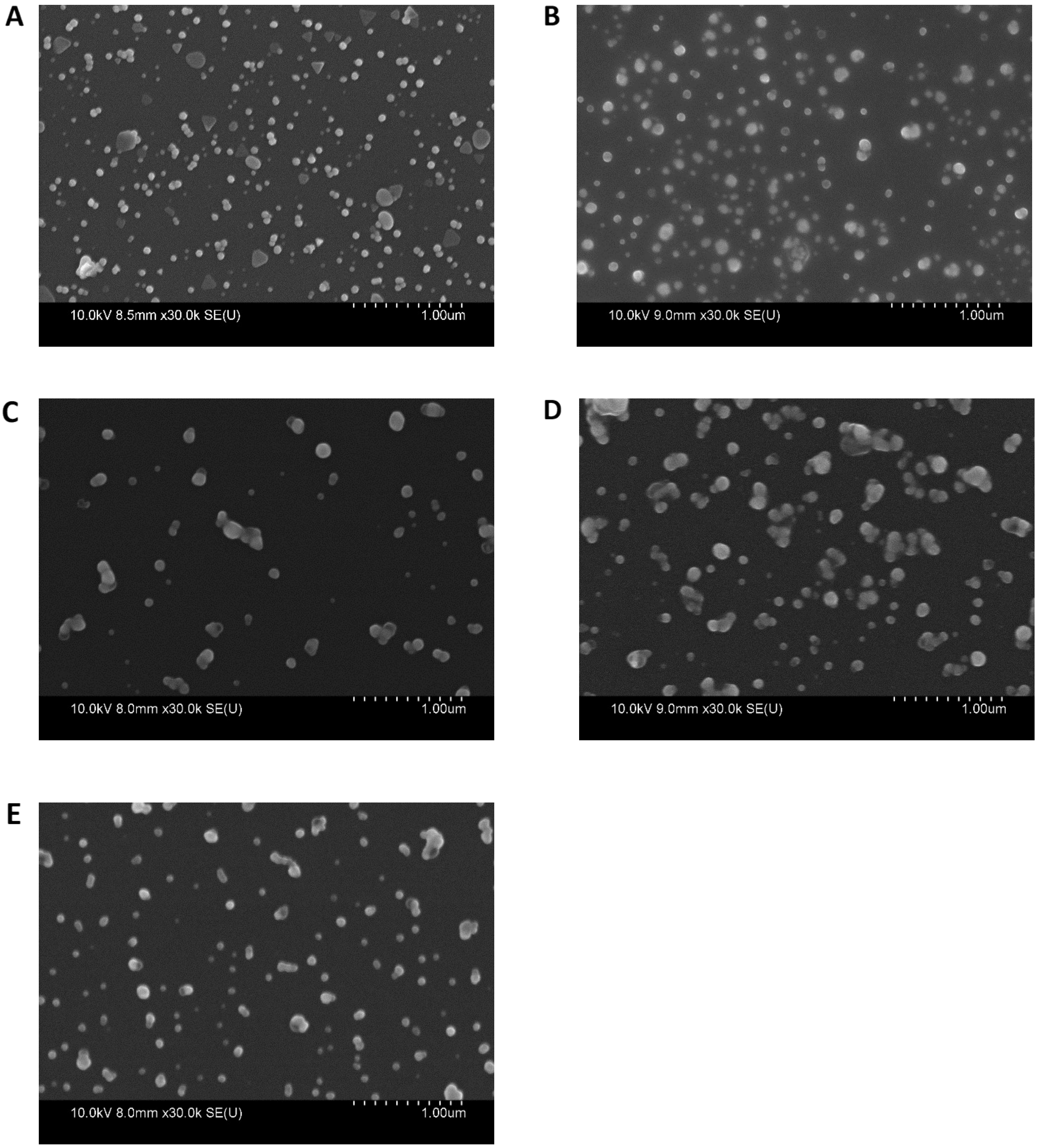

Figure S2: SEM images of **A.** cAuNPs, **B.** hAgNPs batch 1, **C.** hAgNPs batch 2, **D.** hAgNPs batch 3 and **E.** hAgNPs batch 4.

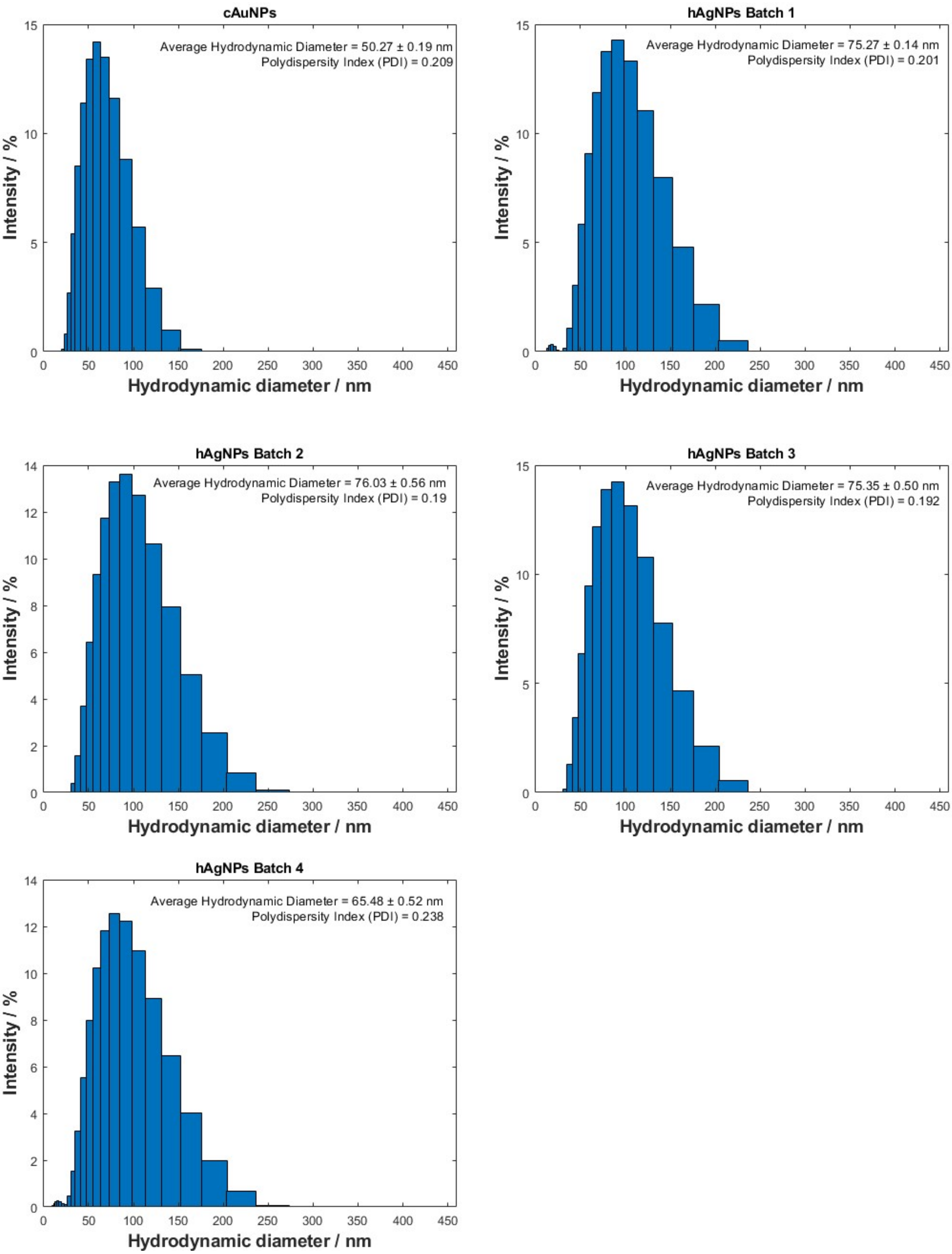

Figure S3: Results from the zetasizer of the average hydrodynamic diameter (n = 5) of citrate stabilised gold nanoparticles (cAuNPs) and different batches of hydroxylamine stabilised silver nanoparticles (hAgNPs). Histograms display size distribution from intensity.

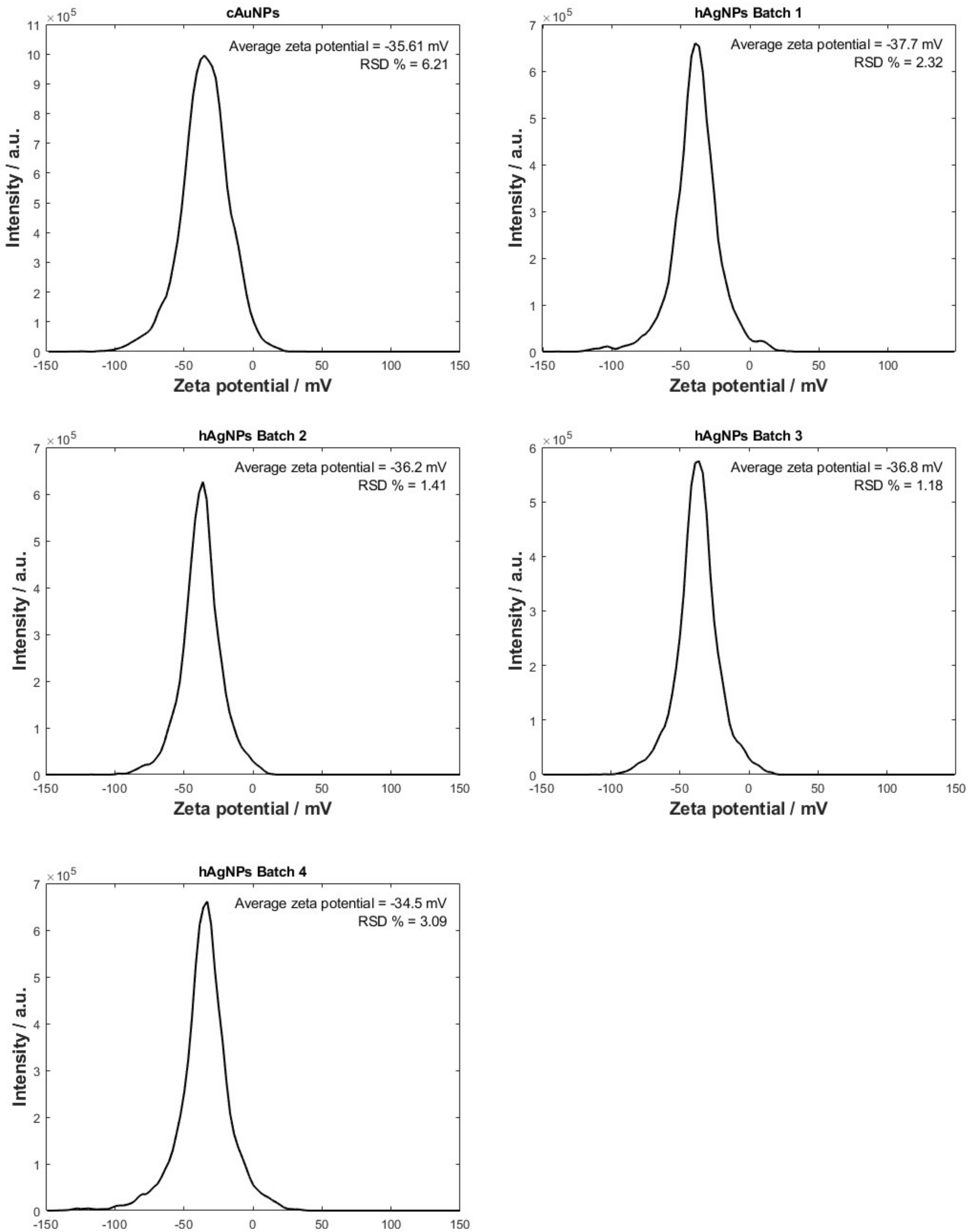

Figure S4: Results from the zetasizer of the average zeta potential (n = 5) of citrate stabilised gold nanoparticles (cAuNPs) and different batches of hydroxylamine stabilised silver nanoparticles (hAgNPs).

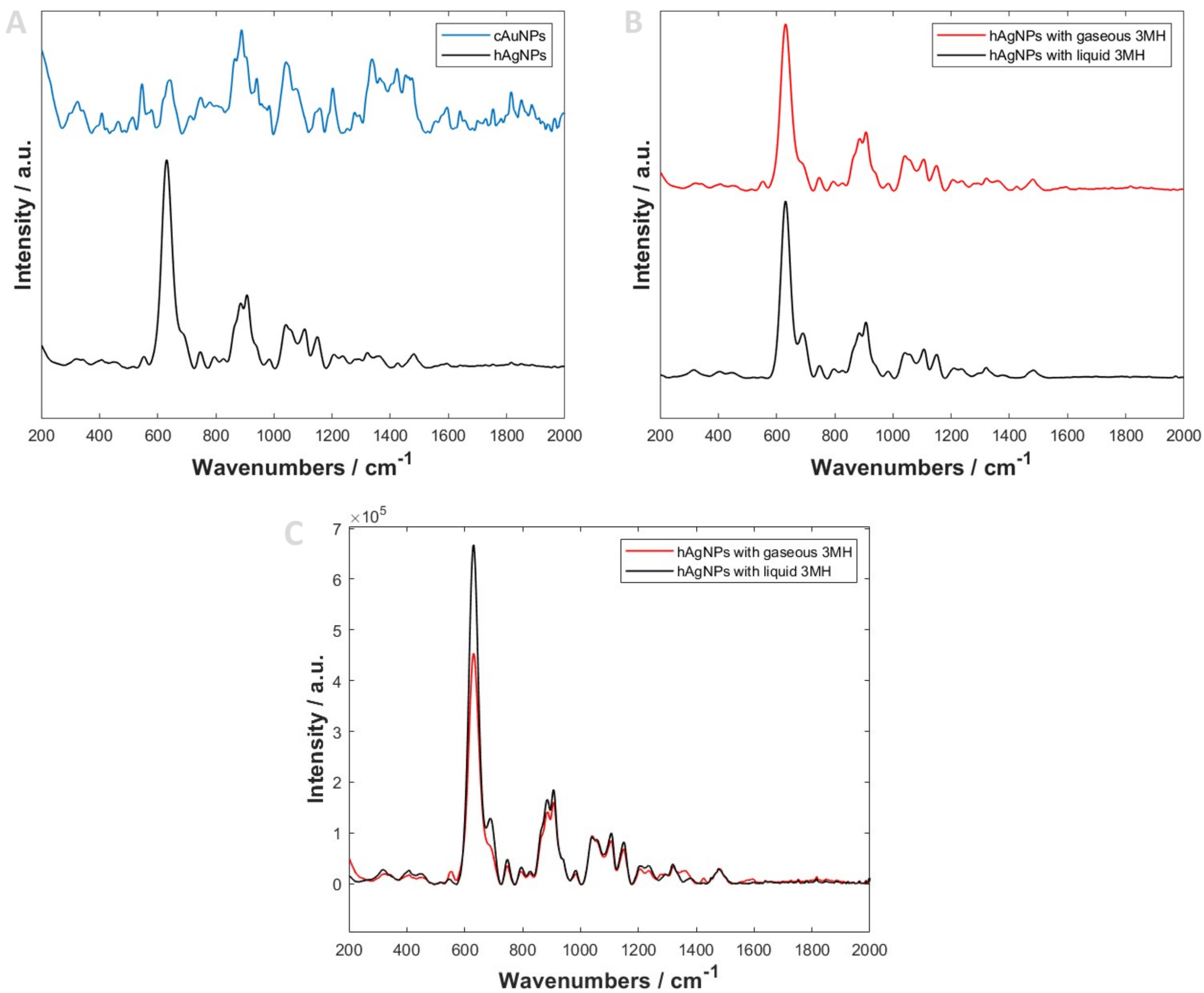

Figure S5: A. Spectra of gaseous 3MH measured using citrate-stabilised gold nanoparticles (cAuNPs) and hydroxylamine-stabilised silver nanoparticles (hAgNPs) 30 min after bubbling 3MH into the nanoparticle solutions. **B.** Comparison of spectra of liquid 3MH (3 ppm) measured with hAgNPs and gaseous 3MH measured with hAgNPs. **C.** Comparison of spectra of liquid 3MH (3 ppm) measured with hAgNPs and gaseous 3MH measured with hAgNPs. The spectra have been baseline corrected and **A.** and **B.** have also been smoothed and vector normalised.

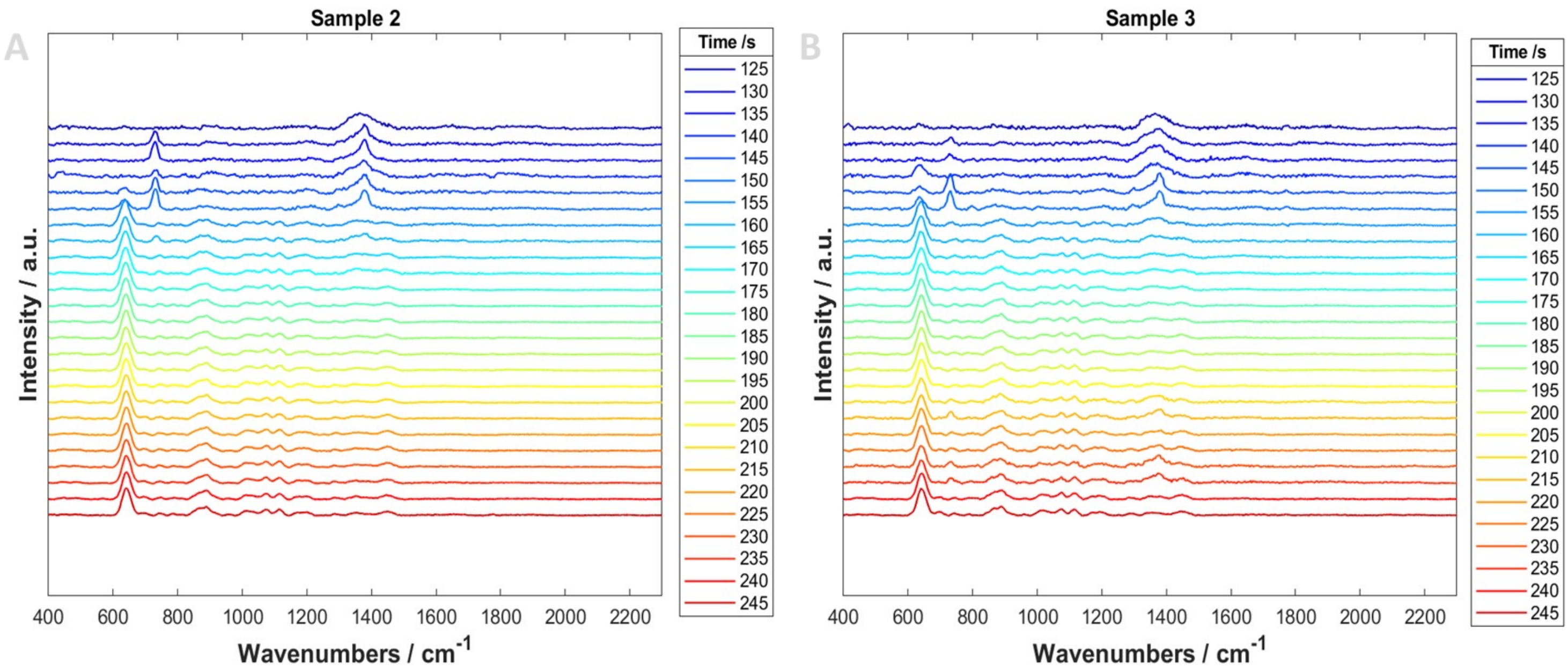

Figure S6: Spectra of 3MH changing from the start (blue) of the bubbling period to the timepoint at which the spectra no longer changes (red) for the **A.** second replicate and **B.** third replicate; with the first replicate shown in **Figure 2**. The kinetic regions was found from the linear regions of the change in peak height at 634 cm^{-1}. The spectra have been baseline corrected, smoothed and vector normalised.

Optimising flow rate

To obtain reproducible results, many experimental parameters can be optimised including temperature, volume of nanoparticles, gas volume and gas flow rate. To demonstrate the impact of adjusting a single parameter, different gas flow rates were tested to see how this affected the speed at which the spectrum of 3MH could initially be detected, the rate of change of intensity of the spectrum of 3MH and to find the flow rate which gives the most reproducible results.

For this experiment, eight different flow rates at 2, 4, 5, 6, 8, 10, 20 and 50 $mL.min^{-1}$ were tested. All other parameters remained constant. Since each flow rate affects how quickly the 20 mL of air is expelled from the syringe, the post-bubbling measurement time was extended from 6 to 15 min. For each flow rate, 3 replicate samples were tested.

To monitor spectral changes over time as 3MH was bubbled into hAgNPs, the peak height at 634 cm^{-1} was calculated for each spectrum. The results for each replicate at each flow rate are shown in **Figure S7**. Firstly, what is noticeable is that higher flow rates led to the earlier appearance of the linear region in the kinetic graph, indicating that the 3MH spectrum was produced sooner. At 2 $mL.min^{-1}$, the first spectrum of 3MH and the peak height at 634 cm^{-1} started to increase at 315 s (5 min 15 s). Whereas at 50 $mL.min^{-1}$, the first spectrum of 3MH appeared and the peak height at 634 cm^{-1} increased at 125 s (2 min 5 s) (**Figure S7 and S8A**). This is likely because a smaller volume of gaseous molecules has been introduced to the nanoparticles at a slower flow rate when comparing the same timepoint at two different flow rates. Hence theoretically, the amount of gaseous 3MH introduced in 5 s at a flow rate of 50 $mL.min^{-1}$ would take approximately 2 min to reach at a flow rate of 2 $mL.min^{-1}$. This results in slower nanoparticle aggregation as less 3MH is interacting with hAgNPs. So, it takes longer for the spectrum of 3MH to be seen. Additionally, faster flow rates enhance mixing due to increased bubble motion, promoting quicker interaction and aggregation.

Furthermore, at slower flow rates from 2 to 10 $mL.min^{-1}$, the peak height increased during bubbling and reached a maximum while 3MH was still being bubbled into hAgNPs. As discussed in the **Results and Discussion,** this implies that 3MH has saturated the nanoparticles causing them all to aggregate so no more molecules could bond to the nanoparticles. This is particularly seen for samples such as at 2 $mL.min^{-1}$ and 4 $mL.min^{-1}$ where some of the scatter plot replicates showed a peak followed by a decrease and then a plateau in peak height across the remaining timeframe (**Figure S7**). The plateau region then suggests that there is a stable spectrum present and there are no further interactions between 3MH and hAgNPs.

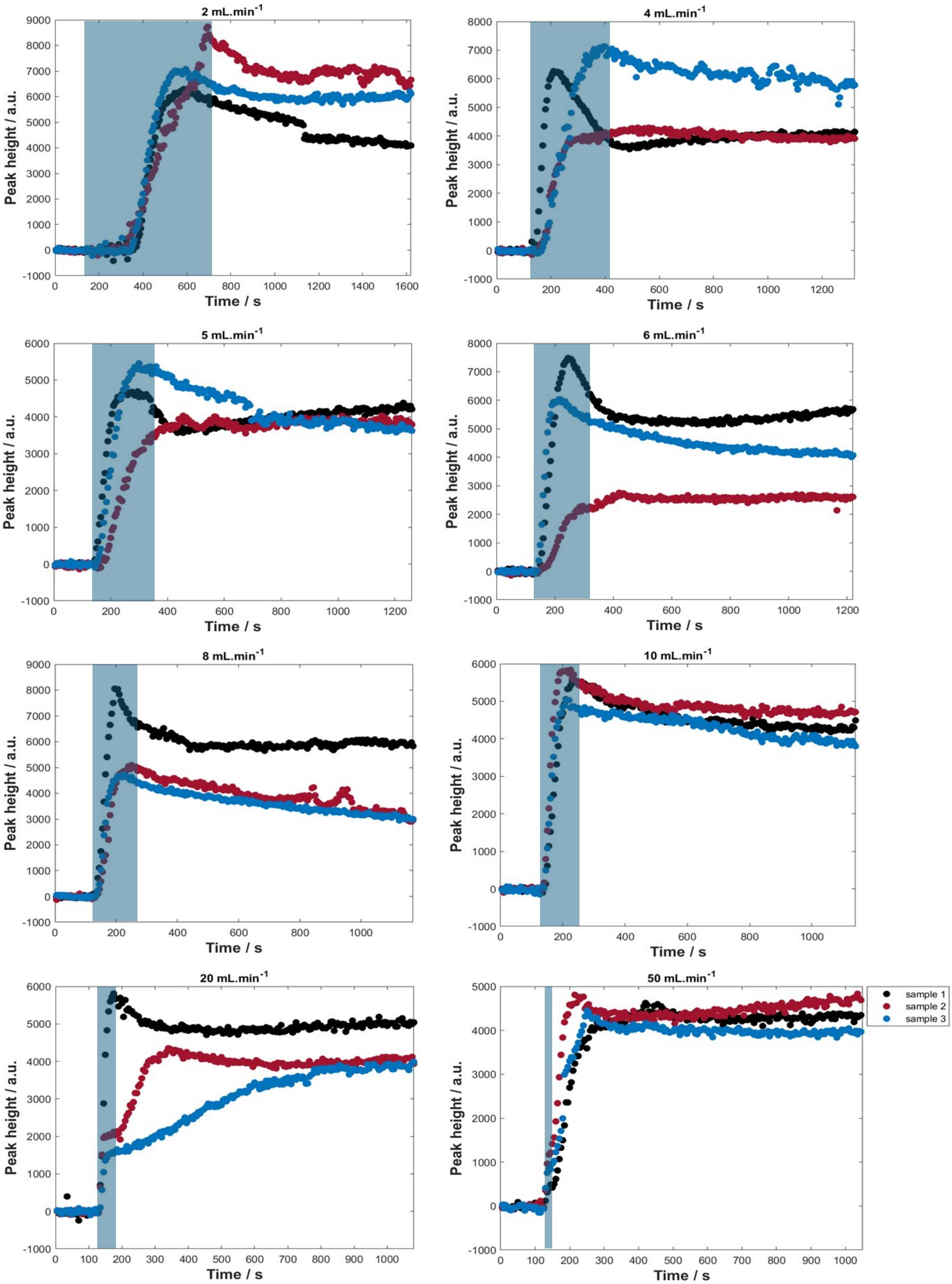

Figure S7: Kinetic graph of changes in peak height at 634 cm^{-1} across the duration of the bubbling experiment at different flow rates between 2 and 50 mL.min^{-1}. The region highlighted in blue represents the timeframe when 3MH was bubbled into hAgNPs.

When the average peak heights are taken across all three replicates for each flow rate and are compared, the peak heights reached plateau regions at similar values across all the flow rates except for 2 mL.min^{-1}, which yielded a noticeably higher average peak height (**Figure S8A**). One possible explanation is that as the thiol is flowing into the nanoparticles at a slower rate, it is interacting with the nanoparticles over a longer period, improving the SERS signal produced. This was further reflected in the rate of change in peak height. The rate of change in peak height was calculated by plotting the linear regions for each replicate at each flow rate. For each replicate at each flow rate, the linear region of the peak height scatter plot was identified, and the gradient of the line, which represents the rate of change, was calculated. The average rate and standard deviation (SD) for each flow rate was then determined from the three replicates. 2 ml min^{-1} showed the slowest rate of change in peak height at 36.42 ± 12.46 a.u. s^{-1} (where 36.42± 12.46 a.u. s^{-1} is the mean rate of change in peak height ± SD) (**Figure S8B** and **Table S1)**. The rate of change then increased, peaking at 8 and 10 mL.min^{-1} with values of 101.12 ± 36.61 a.u. s^{-1} and 97.65 ± 20.43 a.u. s^{-1} respectively, before declining again at higher flow rates. This may be because at faster flow rates, a large amount of gaseous analyte is being bubbled into the colloidal nanoparticles in a short timeframe (a 20 mL syringe is emptied in 24 s at 50 mL.min^{-1}). Consequently, there is greater perturbance to the system over a shorter time period causing slower movements and interactions between all the nanoparticles and analyte molecules after bubbling in 3MH. This slows down the total aggregation of hAgNPs by 3MH in comparison to the other flow rates. While these observations are intriguing, rheological modelling is beyond the scope of this research. Nevertheless, future research could explore the behaviour of gaseous analytes in colloidal systems

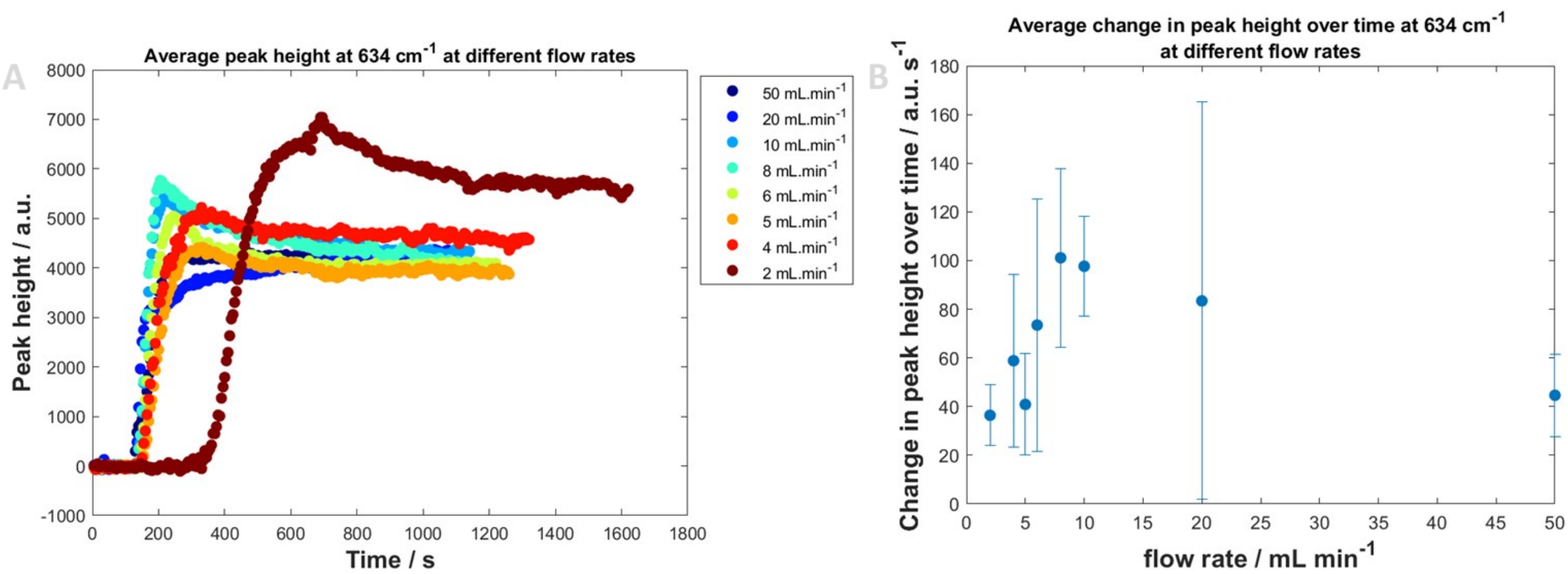

Figure S8: A. Average kinetic graphs from 3 replicate measurements of changes in peak height at 634 cm^{-1} across the duration of the bubbling experiment at different flow rates. **B.** Error plots of average rate of change in peak height at 634 cm^{-1} across the linear region over time. Average rate of change was calculated from the rate of change measured from the linear region of each replicate (n = 3) and error bars represent the standard deviations.

more thoroughly.

Table S1: Average rate of change in peak height at 634 cm^{-1} calculated from the linear region of the kinetic graph of each replicate (n = 3), standard deviations and relative standard deviations (RSD) at the different flow rates tested.

Flow rate ($mL.min^{-1}$)	Average change in peak height at 634 cm^{-1} over time (a.u. s^{-1})	Standard deviation (a.u. s^{-1})	Relative standard deviation (%)
2	36.42	12.46	34.21
4	58.85	35.58	60.46
5	40.88	20.81	50.91
6	73.47	51.99	70.76
8	101.12	36.61	36.20
10	97.65	20.43	20.92
20	83.42	81.62	97.84
50	44.65	16.93	37.92

PCA plots were also produced from all the spectra for each replicate at each timepoint and from the spectra corresponding to the linear region of the 634 cm^{-1} peak height scatter graphs (**Figure 6** and **Figures S9 to S16**). Across all scores plots, a trend was observed in PC-1 from negative to positive score values with increasing time. The loadings plot for PC-1 confirmed that the variation in the spectra over time was driven by the appearance of peaks associated to 3MH. When comparing the linear regions using PCA, the total explained variance (TEV) of PC-2 provided information about the reproducibility of the flow rates and if the final timepoints in the linear region clustered closely together. Among the examined flow rates, 2 $mL.min^{-1}$, 10 $mL.min^{-1}$ and 50 m $L.min^{-1}$ showed the most reproducible results with and PC-1 TEV values of 89.18%, 90.20% and 83.90% and PC-2 TEV values of 3.03%, 3.33% and 3.94%, respectively. The peaks contributing most to the variance in the PC-2 loadings plots at these flow rates were primarily the peak at 634 cm^{-1} and the broad band at 1500 cm^{-1} attributed to the glass background peak. For 50 $mL.min^{-1}$, additional peaks were observed in the PC-2 loadings plot, likely originating from the PTFE tubing in the nanoparticle solution (**Figure S17)**. Further evidence for these three flow rates being the most reproducible are the low RSD values of between 20.92% and 37.92% for the rate of change of peak height, as well as the good overlap between replicate scatter graphs (**Figure S7 and Table S1**).

When the experiments were repeated twice further, a flow rate of 50 $mL.min^{-1}$ showed the most reproducible results (**Figure 3D**). This consistency may be attributed to the slower aggregation of hAgNPs. Unlike at 2 $mL.min^{-1}$, however, the slower aggregation at 50 $mL.min^{-1}$ is likely due to the large amount of 3MH being introduced into the solution of hAgNPs. It is therefore an effect of the mixing of hAgNPs with 3MH. As previously mentioned, faster flow rates result in shorter bubbling durations, leading to rapid and consistent interactions during bubbling. This explains the quick appearance of the 3MH spectrum. This also means that the bubbles no longer have an impact on the Brownian motion of the molecules after the bubbling implying the motion is more consistent across the timeframe of the experiment making technical replicates more reproducible. However, the results from each attempt are different to one another as the length of the two pieces of tubing were being optimised during the other two attempts. The lengths of tubing were longer than for the first attempt which meant the 3MH gas had further to travel to reach the nanoparticles. Therefore, the peak intensity was

lower for the other attempts and the aggregation time was slower. Overall, based on these results 50 mL.min^{-1} was chosen for future measurements of other analytes using the bubbling system.

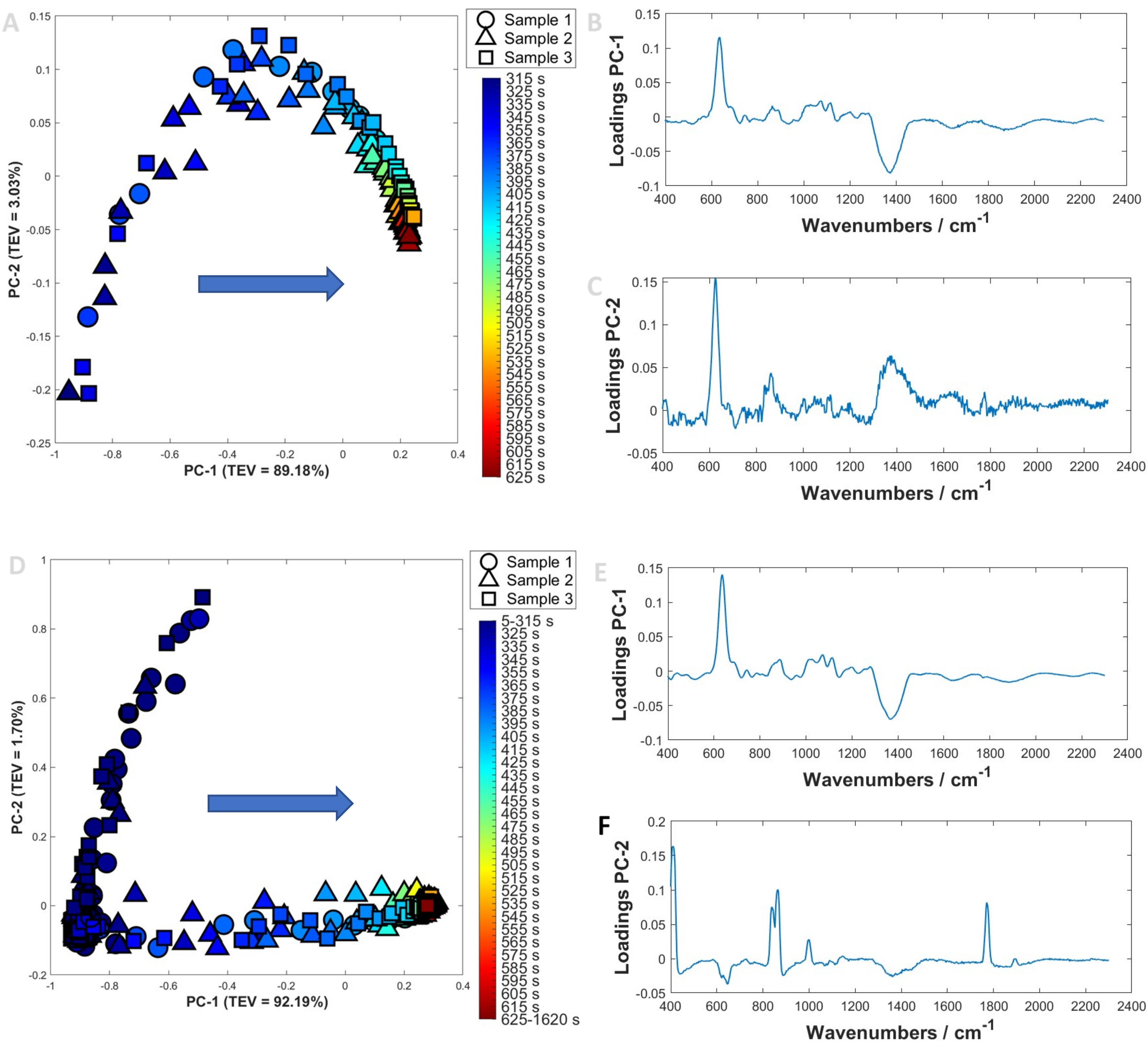

Figure S9: A - C. PCA scores plot and loadings plots for PC-1 and PC-2 of the spectra changing between 315 s (blue) and 625 s (red) during the kinetic region of the bubbling experiment at 2 mL.min^{-1}. **D - F** PCA scores plot and loadings plots for PC-1 and PC-2 of the spectra changing through all timepoints (from 5 s (blue) to 1620 s (red)) of the bubbling experiment at 2 mL.min^{-1}. For **A.** and **D.** The arrow indicates direction of decreasing concentration and TEV is the total explained variance. Spectra has been baseline corrected, smoothed and vector normalised.

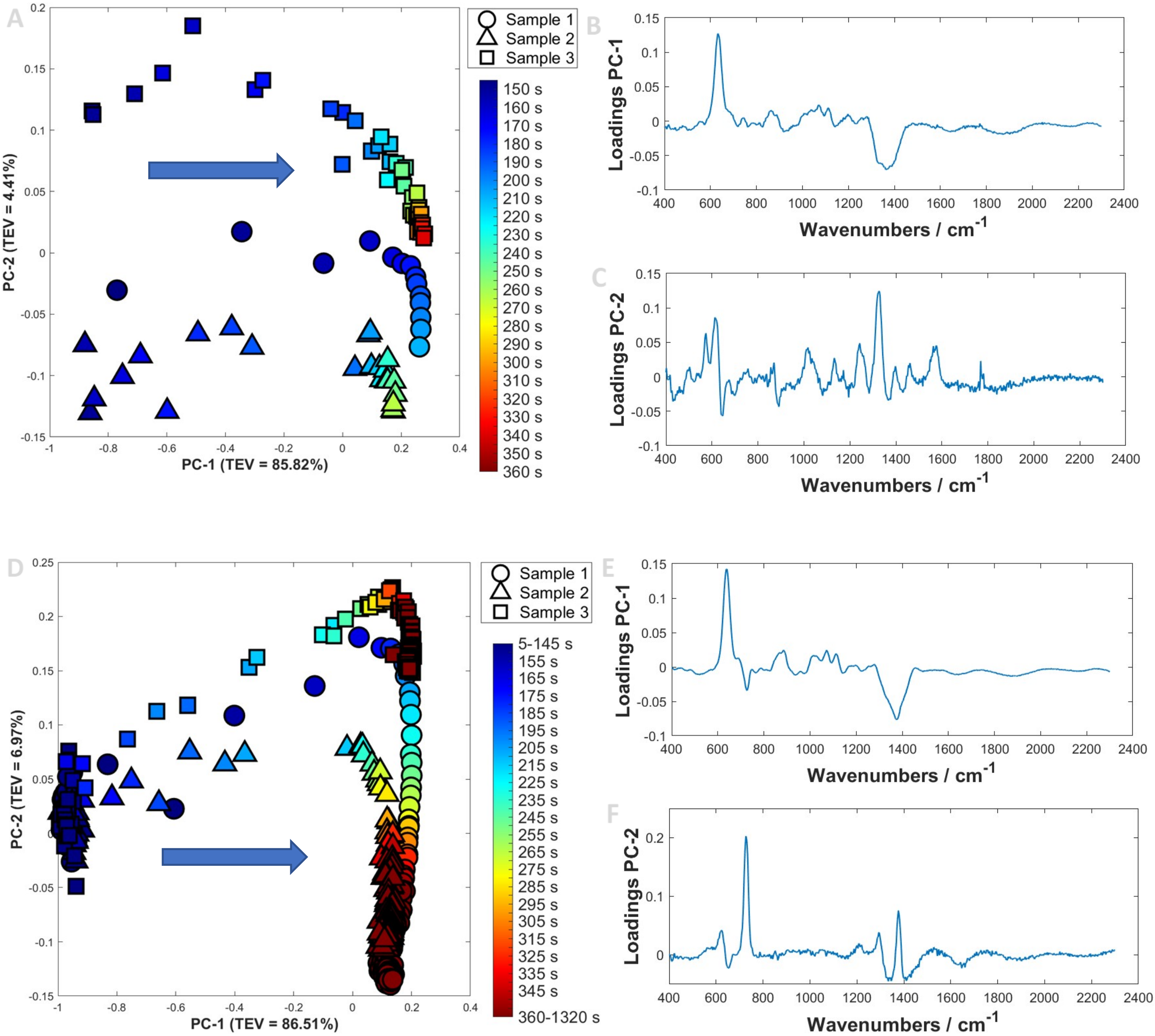

Figure S10: A - C. PCA scores plot and loadings plots for PC-1 and PC-2 of the spectra changing between 145 s (blue) and 360 s (red) during the kinetic region of the bubbling experiment at 4 mL.min^{-1}. **D - F** PCA scores plot and loadings plots for PC-1 and PC-2 of the spectra changing through all timepoints (from 5 s (blue) to 1320 s (red)) of the bubbling experiment at 4 mL.min^{-1}. For **A.** and **D.** The arrow indicates direction of decreasing concentration and TEV is the total explained variance. Spectra has been baseline corrected, smoothed and vector normalised.

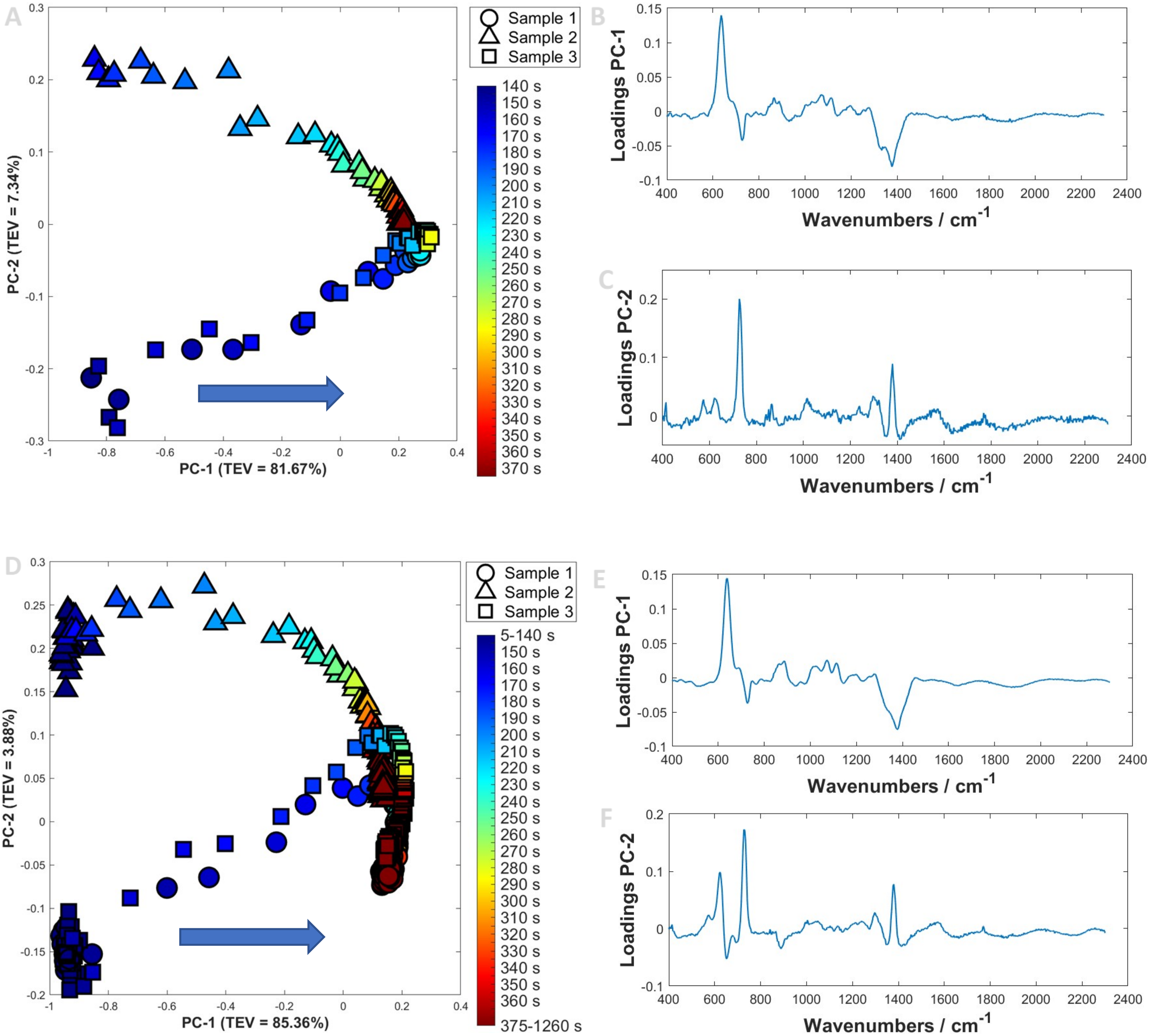

Figure S11: A - C. PCA scores plot and loadings plots for PC-1 and PC-2 of the spectra changing between 140 s (blue) and 375 s (red) during the kinetic region of the bubbling experiment at 5 mL.min^{-1}. **D - F** PCA scores plot and loadings plots for PC-1 and PC-2 of the spectra changing through all timepoints (from 5 s (blue) to 1260 s (red)) of the bubbling experiment at 5 mL.min^{-1}. For **A.** and **D.** The arrow indicates direction of decreasing concentration and TEV is the total explained variance. Spectra has been baseline corrected, smoothed and vector normalised.

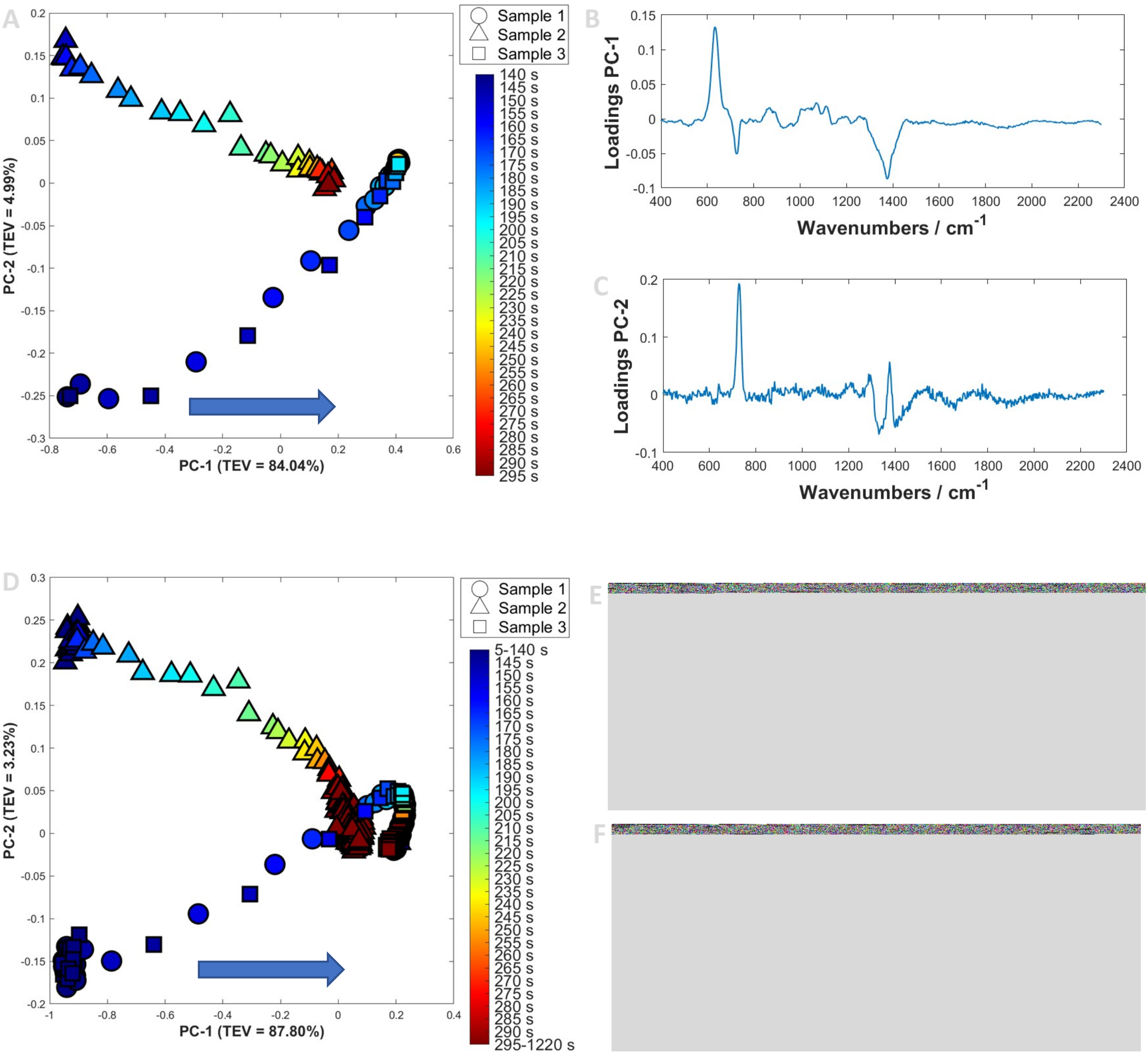

Figure S12: A - C. PCA scores plot and loadings plots for PC-1 and PC-2 of the spectra changing between 140 s (blue) and 295 s (red) during the kinetic region of the bubbling experiment at 6 mL.min^{-1}. **D - F** PCA scores plot and loadings plots for PC-1 and PC-2 of the spectra changing through all timepoints (from 5 s (blue) to 1220 s (red)) of the bubbling experiment at 6 mL.min^{-1}. For **A.** and **D.** The arrow indicates direction of decreasing concentration and TEV is the total explained variance. Spectra has been baseline corrected, smoothed and vector normalised.

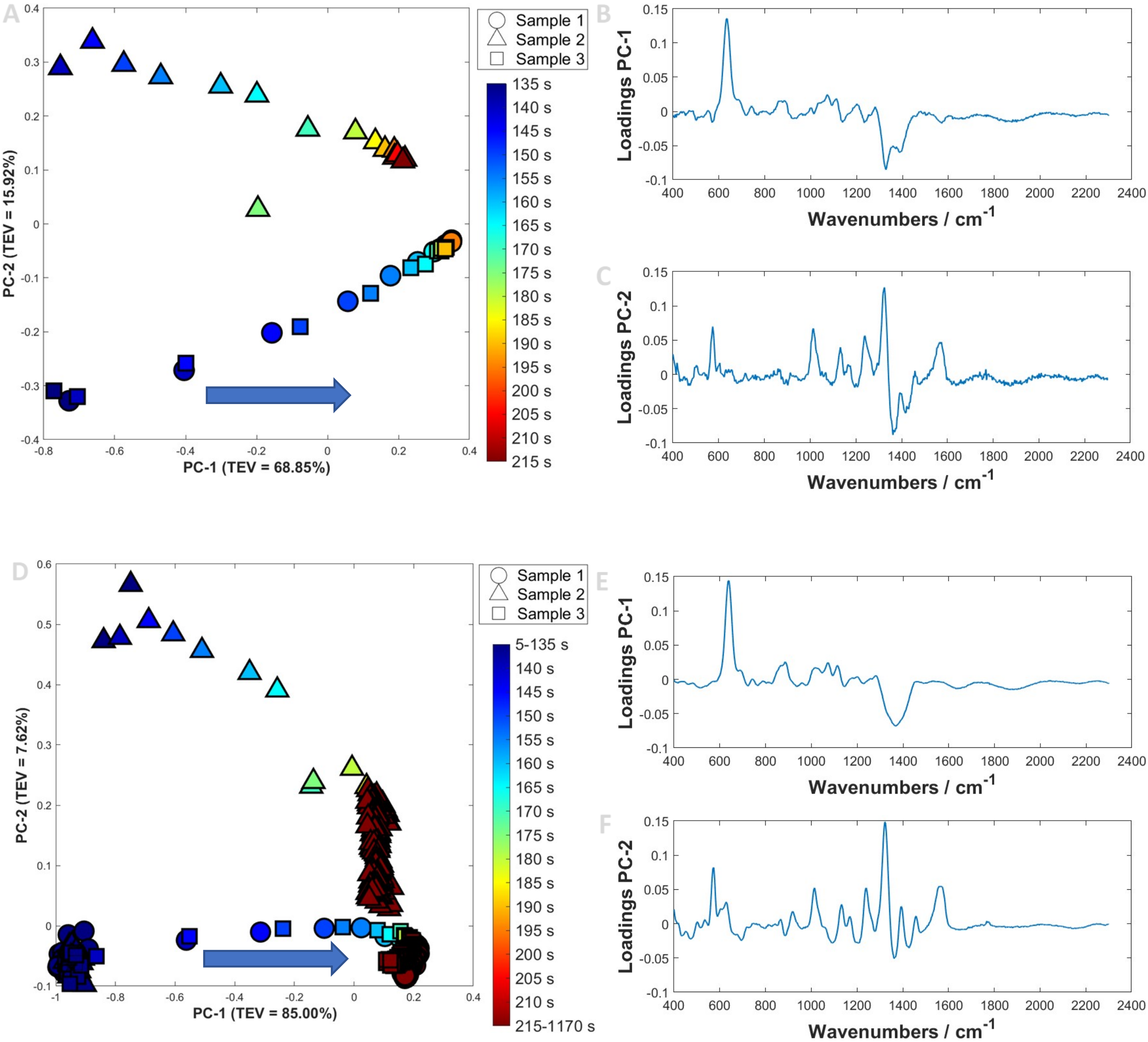

Figure S13: A - C. PCA scores plot and loadings plots for PC-1 and PC-2 of the spectra changing between 135 s (blue) and 215 s (red) during the kinetic region of the bubbling experiment at 8 mL.min^{-1}. **D - F** PCA scores plot and loadings plots for PC-1 and PC-2 of the spectra changing through all timepoints (from 5 s (blue) to 1170 s (red)) of the bubbling experiment at 8 mL.min^{-1}. For **A.** and **D.** The arrow indicates direction of decreasing concentration and TEV is the total explained variance. Spectra has been baseline corrected, smoothed and vector normalised.

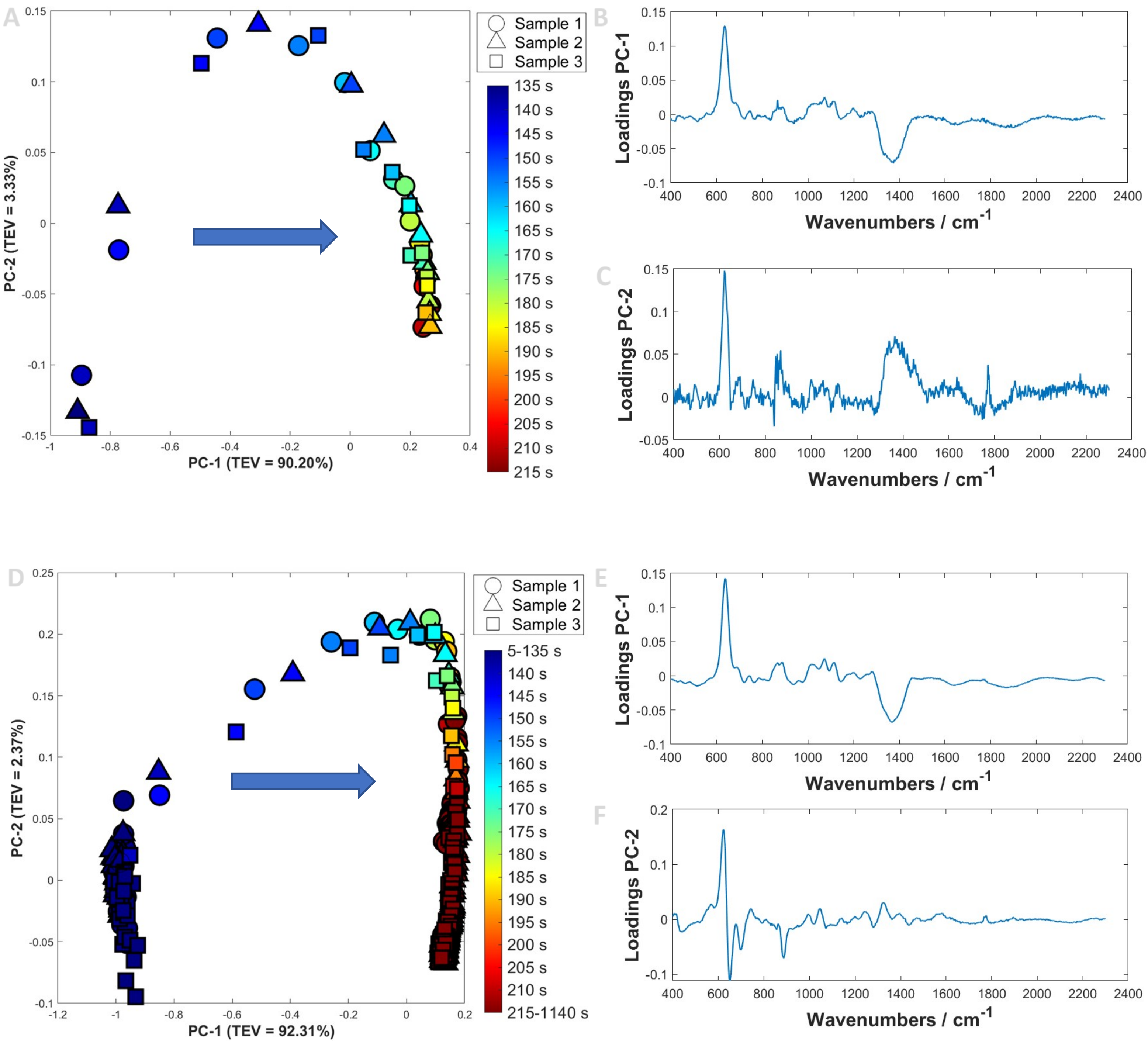

Figure S14: A - C. PCA scores plot and loadings plots for PC-1 and PC-2 of the spectra changing between 135 s (blue) and 215 s (red) during the kinetic region of the bubbling experiment at 10 mL.min^{-1}. **D - F** PCA scores plot and loadings plots for PC-1 and PC-2 of the spectra changing through all timepoints (from 5 s (blue) to 1140 s (red)) of the bubbling experiment at 10 mL.min^{-1}. For **A.** and **D.** The arrow indicates direction of decreasing concentration and TEV is the total explained variance. Spectra has been baseline corrected, smoothed and vector normalised.

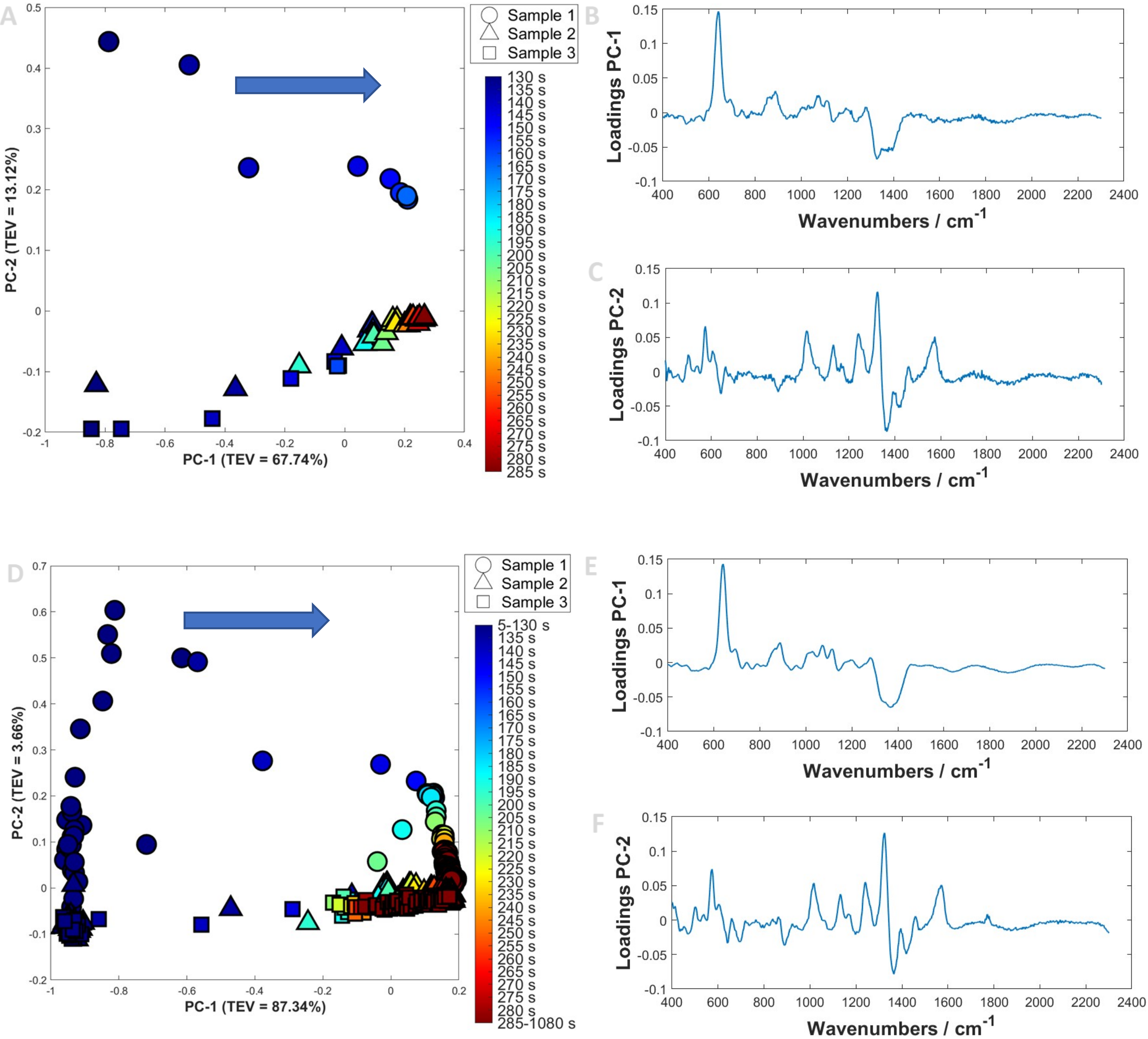

Figure S15: A - C. PCA scores plot and loadings plots for PC-1 and PC-2 of the spectra changing between 130 s (blue) and 285 s (red) during the kinetic region of the bubbling experiment at 20 mL.min^{-1}. **D - F** PCA scores plot and loadings plots for PC-1 and PC-2 of the spectra changing through all timepoints (from 5 s (blue) to 1080 s (red)) of the bubbling experiment at 20 mL.min^{-1}. For **A.** and **D.** The arrow indicates direction of decreasing concentration and TEV is the total explained variance. Spectra has been baseline corrected, smoothed and vector normalised.

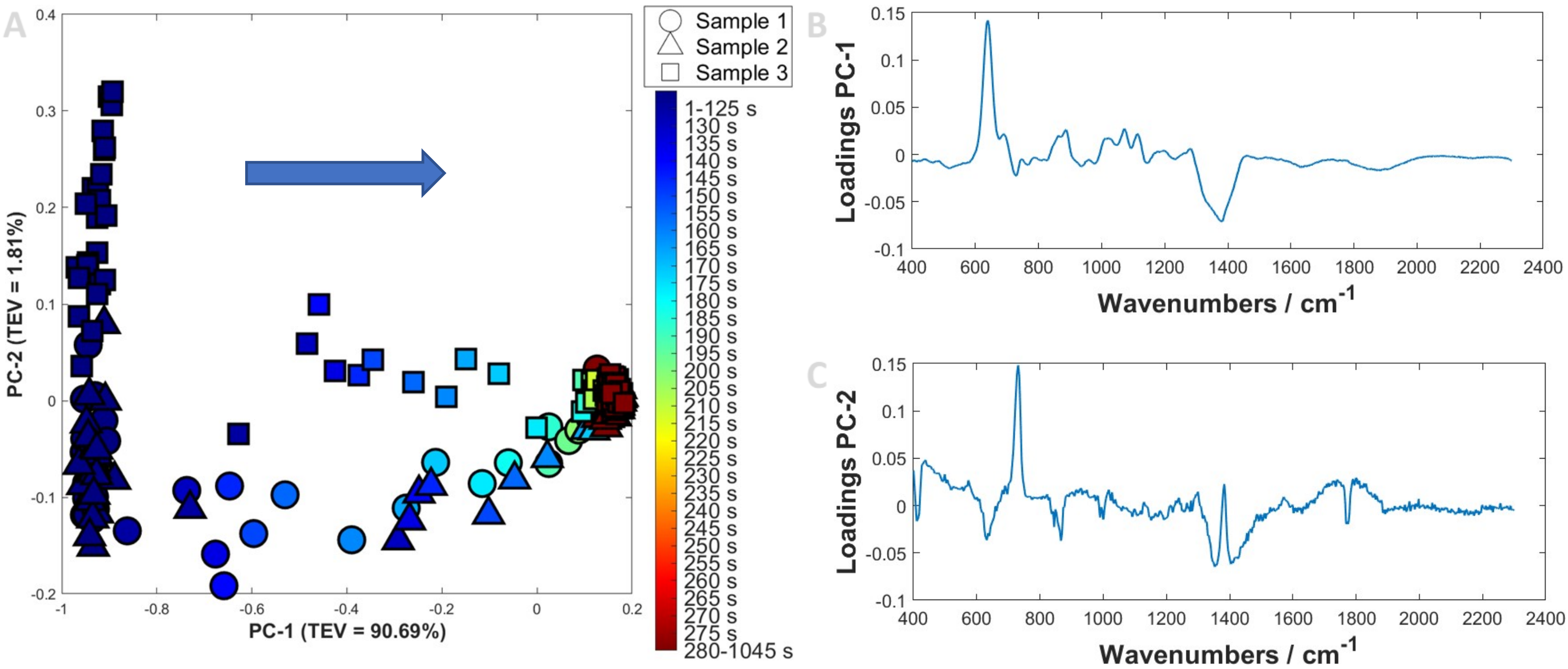

Figure S16: A. PCA scores plot of the spectra changing through all timepoints (from 5 s (blue) to 1045 s (red)) of the bubbling experiment at 50 ml.min^{-1}. The arrow indicates direction of decreasing concentration and TEV is the total explained variance. **B.** Loadings plot of PC-1 for all timepoints in the kinetic experiment at 50 mL.min^{-1}. **C.** Loadings plot of PC-2 for all timepoints in the kinetic experiment at 50 mL.min^{-1}. Spectra has been baseline corrected, smoothed and vector normalised.

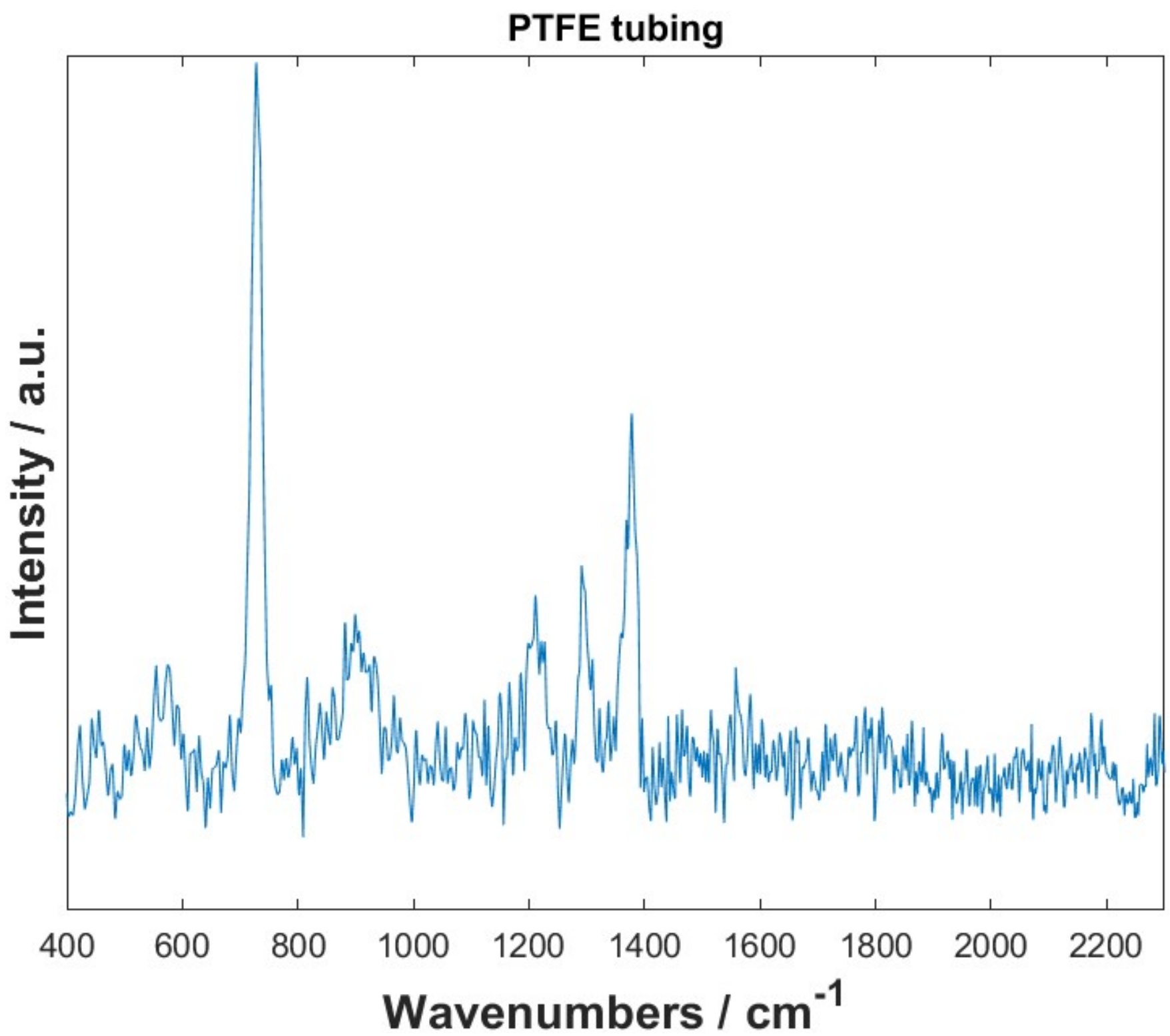

Figure S17: Raman spectrum of PTFE tubing used for transporting the gaseous analyte into the vial of metallic nanoparticles. Spectra has been baseline corrected, smoothed and vector normalised.

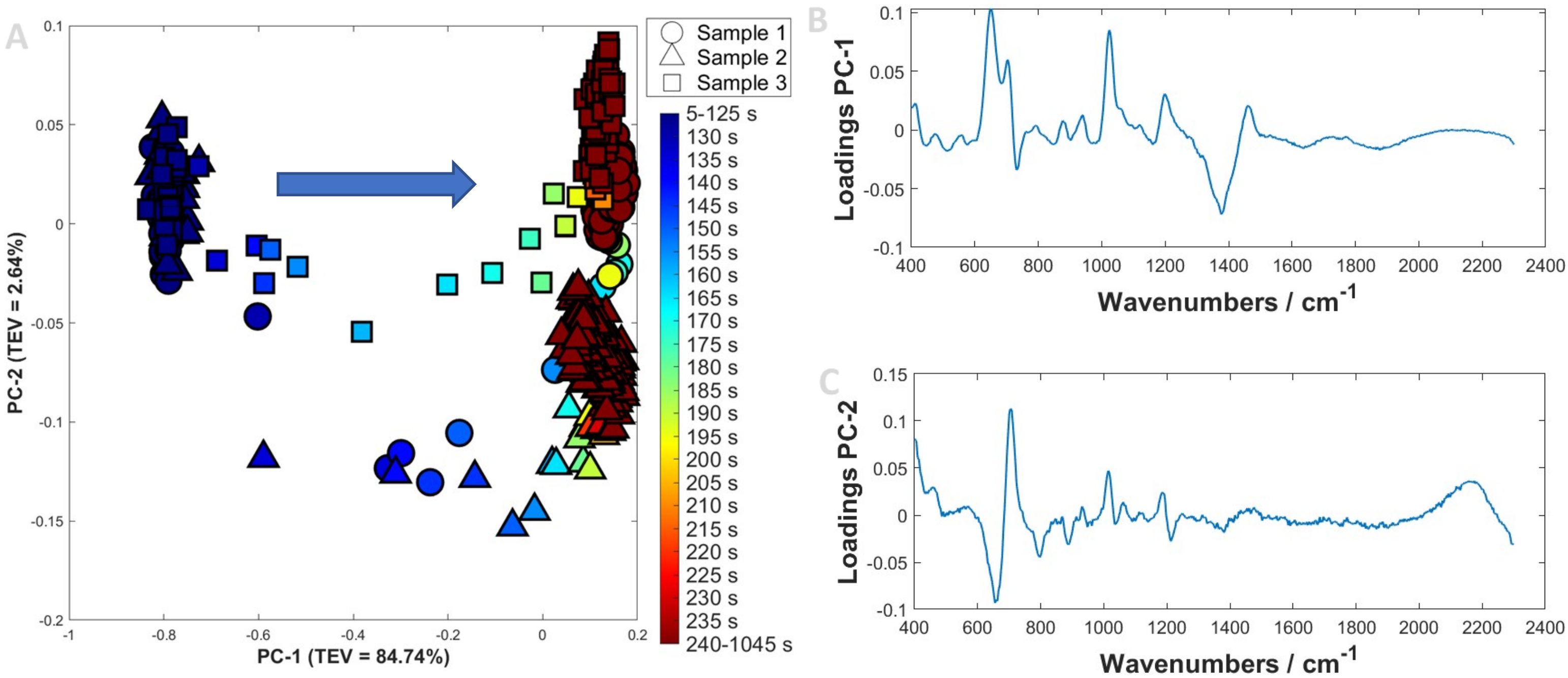

Figure S18: A. PCA scores plot of the spectra for 2-methyl-3-mercapto-pentan-1-ol (2M3MP) changing through all timepoints (from 5 s (blue) to 1045 s (red)) of the bubbling experiment at 50 mL.min^{-1}. The arrow indicates direction of decreasing concentration and TEV is the total explained variance. **B.** Loadings plot of PC-1 for all timepoints in the kinetic experiment at 50 mL.min^{-1}. **C.** Loadings plot of PC-2 for all timepoints in the kinetic experiment at 50 mL.min^{-1}. Spectra has been baseline corrected, smoothed and vector normalised.

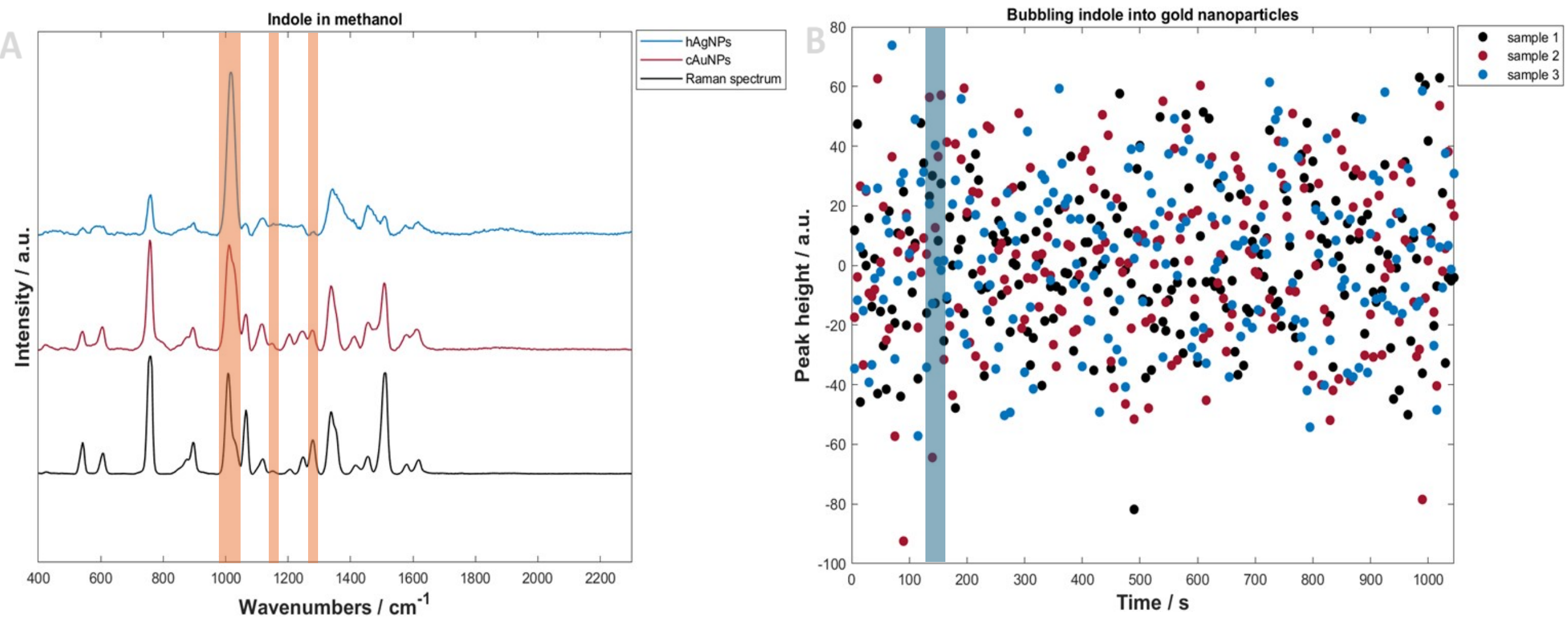

Figure S19: A. Raman spectrum of indole in methanol and SERS spectra of indole in methanol produced using hAgNPs and cAuNPs. Peaks highlighted in orange represent peaks found in the spectrum of methanol. The spectra have been baseline corrected, smoothed and vector normalised. **B.** Kinetic graph of changes in peak height at 760 cm^{-1} for indole across the duration of the bubbling experiment at 50 mL.min^{-1} for the three replicate measurements. The region highlighted in blue represents the timeframe when indole was bubbled into cAuNPs.

References

1. Leopold, N.; Lendl, B., A New Method for Fast Preparation of Highly Surface-Enhanced Raman Scattering (SERS) Active Silver Colloids at Room Temperature by Reduction of Silver Nitrate with Hydroxylamine Hydrochloride. *The Journal of Physical Chemistry B* **2003,** *107* (24), 5723-5727.
2. Lee, P. C.; Meisel, D., Adsorption and surface-enhanced Raman of dyes on silver and gold sols. *The Journal of Physical Chemistry* **1982,** *86* (17), 3391-3395.

3. Dong, X.; Gu, H.; Kang, J.; Yuan, X.; Wu, J., Comparative study of surface-enhanced Raman scattering activities of three kinds of silver colloids when adding anions as aggregating agents. *Colloids and Surfaces A: Physicochemical and Engineering Aspects* **2010,** *368* (1), 142-147.
4. Mikac, L.; Ivanda, M.; Gotić, M.; Mihelj, T.; Horvat, L., Synthesis and characterization of silver colloidal nanoparticles with different coatings for SERS application. *J. Nanopart. Res.* **2014,** *16* (12), 2748.
